# ALA induces stomatal opening through regulation among PTPA, PP2AC, and SnRK2.6

**DOI:** 10.3389/fpls.2023.1206728

**Published:** 2023-08-30

**Authors:** Zheng Chen, Jianting Zhang, Liangju Wang

**Affiliations:** College of Horticulture, Nanjing Agricultural University, Nanjing, China

**Keywords:** 5-aminolevulinic acid, *Malus domestica*, MdPP2AC, MdPTPA, MdSnRK2.6, *Nicotiana tabacum*, stomatal movement

## Abstract

5-Aminolevulinic acid (ALA), as a new natural plant growth regulator, has been proved to regulate protein phosphatase 2A (PP2A) activity to promote stomatal opening in apple (*Malus domestica*) leaves. However, the molecular mechanisms underlying remain unclear. Here, we cloned and transformed *MdPTPA*, *MdPP2AC*, and *MdSnRK2.6* of apple into tobaccos (*Nicotiana tabacum*) and found that over-expression (OE)-*MdPTPA* or OE-*MdPP2AC* promoted stomatal aperture while OE-*MdSnRK2.6* induced stomatal closure under normal or drought condition. The Ca^2+^ and H_2_O_2_ levels in the guard cells of OE-*MdPTPA* and OE-*MdPP2AC* was decreased but flavonols increased, and the results in OE-*SnRK2.6* was contrary. Exogenous ALA stimulated PP2A activity but depressed SnRK2.6 activity in transgenic tobaccos, leading to less Ca^2+^, H_2_O_2_ and more flavonols in guard cells, and consequently stomatal opening. OE-*MdPTPA* improved stomatal opening and plant growth but impaired drought tolerance, while OE-*MdSnRK2.6* improved drought tolerance but depressed the leaf *P*_n_. Only OE-*MdPP2AC* improved stomatal opening, leaf *P*_n_, plant growth, as well as drought tolerance. These suggest that the three genes involved in ALA-regulating stomatal movement have their respective unique biological functions. Yeast two-hybrid (Y2H) assays showed that MdPP2AC interacted with MdPTPA or MdSnRK2.6, respectively, but no interaction of MdPTPA with MdSnRK2.6 was found. Yeast three-hybrid (Y3H) assay showed that MdPTPA promoted the interactions between MdPP2AC and MdSnRK2.6. Therefore, we propose a regulatory module of PTPA-PP2AC-SnRK2.6 that may be involved in mediating the ALA-inducing stomatal aperture in green plants.

## Introduction

Stomata are the main gateway for plants to communicate with the environment. Water loss through transpiration, atmospheric CO_2_ entrance needed by photosynthesis and O_2_ release into atmosphere are all mainly controlled and modulated by stomatal aperture. They are the “sentinels” of plants, ambushing on the underside of plant leaves, watching the diurnal changes of the surrounding environments, responding to light, temperature, humidity, and atmosphere to control gas exchange and modulate water balance of plants. The extent of stomatal opening is closely related to leaf photosynthesis, plant activities and agricultural production (Murata et al., 2015). Study on stomatal movement and its regulation mechanism is of great scientific significance and practical value, which is always a hotspot in plant science and agricultural studies (Wang et al., 2004; Murata et al., 2015).

5-Aminolevulinic acid (ALA), as the key precursor for biosynthesis of all tetrapyrrole compounds such as chlorophyll and heme, has been suggested to be a new plant growth regulator (Wang et al., 2003; Akram and Ashraf, 2013; Wu et al., 2019). In stomatal aperture, Wang et al. (2004) firstly reported that ALA had a function to promote stomatal opening in melon leaves, which has been ascribed to be an important mechanism for ALA to enhance plant photosynthesis. Up to now, ALA is known to reverse the stomatal closure induced by drought stress (Al-Khateeb, 2006), exogenous abscisic acid (ABA) or darkness (Chen et al., 2014b), where hydrogen peroxide (H_2_O_2_) and calcium ion (Ca^2+^) are depressed (An et al., 2016b) while flavonols are significantly accumulated in guard cells. All these changes can be observed in ALA-ABA regulated stomatal movement (An et al., 2016a; Liu et al., 2016). Recently, ALA-induced stomatal aperture has been ascribed to its inducing PP2A (protein phosphatase 2A) activity increase and guard cell microtubule polymerization in apple (Xiong et al., 2018) and Arabidopsis (An et al., 2020). These results provide a new sight on the improvement of plant photosynthesis by ALA.

PP2A, as a trimeric protein complex, composed of scaffold subunit A, regulatory subunit B and catalytic subunit C (Janssens and Goris, 2001), is one of the major serine/threonine protein phosphatases (DeLong, 2006; Farkas et al., 2007). In Arabidopsis, PP2A is known to be an important regulatory factor for ABA signaling (Kwak et al., 2002; Pernas et al., 2007; Saito et al., 2008), however, different subunits of the holoenzyme exhibit different functions. For example, *Atpp2aa1* mutant greatly impaired the ABA sensitivity of seed germination and stomatal movement, suggesting that PP2AA1 is a positive regulator of ABA signal transduction (Saito et al., 2008). Conversely, *Atpp2ac2* mutant was more sensitive to ABA signals during seed germination, root growth, and seedling development, indicating that PP2AC2 plays a negative role in regulation of ABA signal transduction (Pernas et al., 2007). Overexpression of *AtPP2AC-5* promoted the PP2A activity and conferred better growth of root and shoot of *Arabidopsis* under salt condition (Hu et al., 2017). Our previous pharmacological and genetic studies with detached apple leaves showed that *MdPP2AC* was involved in ALA-ABA modulating stomatal movement (Chen et al., 2023). In the regulation, MdPP2AC is positive for ALA signaling but negative for ABA signaling. Furthermore, PTPA, a phosphotyrosyl phosphatase activator, has been suggested to be depended by the assembly of PP2A holoenzymes, since it can promote the methylation of the catalytic C-subunit of PP2A, triggering PP2A activity and negatively regulating ABA signaling (Chen et al., 2014a). Nevertheless, PTPA had been considered as one of B subunits of PP2A because it bound to the AC dimers of the PP2A holoenzyme (Fellner et al., 2003). Recently, we demonstrated that PTPA participated in ALA-ABA modulating stomatal movement by improving PP2A activity of detached apple leaves (Chen and Wang, 2022).

SRK2E/OST1/SnRK2.6 is a key positive factor of the ABA signaling pathway (Mustilli et al., 2002; Li et al., 2021). SnRK2.6 phosphorylation and dephosphorylation controls stomatal closing and opening (Mustilli et al., 2002; Yoshida et al., 2002). In the previous study, we demonstrated that SnRK2.6 functioned at the downstream of PP2A signaling, involved in ALA-ABA regulation of apple stomatal movement (Chen et al., 2023). Based on these studies, we hypothesized that ALA positively regulated *PTPA* and *PP2AC* expression to enhance PP2A activity, and then negatively regulated SnRK2.6 activity, which finally promoted stomatal opening and improved leaf photosynthetic efficiency of plants. However, the hypothesis was verified in the detached apple leaves rather than living plants. More biological functions of the genes need to be explored.

In current study, we transformed the *MdPTPA*, *MdPP2AC* and *MdSnRK2.6* of apple into tobacco, then compared the photosynthetic gas exchange characteristics and drought tolerance among the transgenic plants. The results showed that overexpression (OE) of *MdPTPA* promoted stomatal aperture but impaired drought tolerance, while OE-*MdSnRK2.6* kept drought tolerance but with less stomatal aperture and photosynthesis. Only OE-*MdPP2AC* exhibited both higher photosynthesis and drought tolerance. Our research reveals new physiological and ecological functions of genes involved in ALA signal transduction in guard cells and provides new insights into the regulation of stomatal movement. Additionally, we found that PTPA-PP2AC-SnRK2.6 interacted together, where PP2AC interacted with PTPA and SnRK2.6, respectively, but PTPA did not directly interact with SnRK2.6. Therefore, we propose a new regulatory module of PTPA-PP2AC-SnRK2.6, which may be involved in mediating the ALA-inducing stomatal aperture in green plants.

## Materials and methods

### Constructions of transformation vectors and selection of transgenic tobaccos

The cDNA fragments of *MdPTPA*, *MdPP2AC* and *MdSnRK2.6* with stop codons based on apple (*Malus domestica*) genome were respectively cloned by polymerase chain reaction (PCR) and ligated into PBI121 using the XbaI and BamHI sites for *GUS* fusion. The constructions of transformation vectors PBI121-*MdPTPA*-*GUS*, PBI121-*MdPP2AC*-*GUS*, PBI121-*MdSnRK2.6*-*GUS* were transformed into *Agrobacterium tumefaciens* GV3101 (Shanghai Weidi Biotechnology Co., Ltd, CAT#: AC1001), respectively. The leaf disc method (Horsch et al., 1985) was used to induce transgenic tobacco (*Nicotiana tabacum*) plants. During genetic transformation, the leaf discs were infected by the reconstructed Agrobacterium and co-cultured for the T-DNA transfer into plant cells. Then they were cultured on the screening MS media with 100 mg L^-1^ kanamycin to induce resistant calli and adventitious buds in a growth chamber at 25°C with 16/8 h light/dark cycle (**Figure S1A**). Three weeks later, the regenerated shoots were induced to root and detected by PCR, GUS, and RT-qPCR methods to identify positive transgenic seedlings.

When PCR detection was conducted, the upstream primers were designed on a 35S promoter (GACGCACAATCCCACTATCC), and downstream primers were designed with *MdPTPA*, *MdPP2AC*, *MdSnRK2.6* genes, respectively (**Table S1**). Eight lines were detected, among them, at least 6 were positive transgenic plants (**Figure S1B**), showing that the target genes were transformed into the tobacco chromosome.

When GUS dying assay was conducted, the epidermal of transgenic tobacco plant leaves were submerged into the GUS staining solution (100 mM sodium phosphate, pH 7, 0.1% (v/v) Triton X-100, 10 mM EDTA, 0.5 mM K_3_Fe(CN)_6_, 0.5 mM K_4_Fe(CN)_6_, and 1 mM 5-bromo-4-chloro-3-indolyl glucuronide) and kept at 37°C for 24 h before removing the chlorophylls with 95% (v/v) ethanol. Images of epidermal cells were captured with the Nikon D50 6.1 MP Digital SLR Camera. When guard cells were stained blue, the plants were judged as the transgenic lines (**Figure S1C**).

When RT-qPCR was conducted, RNA extraction and reverse transcription experiments were performed using kits (Cat. No 4992858; Cat. No AE341-02, Trans Gen Biotech, China), respectively, following the manufacturer protocols. Primer 5 software was used to design primers for real-time PCR, with *Ntβ-actin* (AACCCCTTGTCTGTGATAACG, TCCTTTTGACCCATACCAACC) gene as the internal reference gene. RT-qPCR reaction system (20 μL) consisted of 10 μL SYBR Premix ExTaq, 0.4 μL forward specific primer (10 μM), 0.4 μL reverse specific primer (10 μM), 2 μL cDNA (100 ng μL^-1^) template, and 7.2 μL ddH_2_O. And the reactions were performed using Quant Studio 6Flex (ABI), with the procedures as follows: pre-denaturation at 95°C for 2 min, denaturation at 95°C for 15 s, annealing at 60°C for 20 s, 40 cycles. The primer sequences used are listed in **Table S1**. The 2^−ΔΔCT^ method was used to calculate the relative expressions of genes (Livak and Schmittgen, 2010), and each gene was biologically repeated 3 times. The primer sequences used are listed in **Table S1**. The identified positive transgenic lines were further cultured to produce homozygous offspring by self-pollination.

### Plant materials and chemical treatments

The seeds of homozygous T_2_-generation transgenic tobacco obtained from the above experiments were seeded on 1/2 MS medium at 22°C, 70% relative humidity, and 16/8 h (day/night) photoperiod. About 30 days later, the plants were transplanted into a 20 cm × 20 cm pot and cultured in a growth chamber (23-27°C, 10 h light/14 h dark). When the seedling height was about 20 cm, plants with consistent vegetative growth were selected for the next experiment. The epidermis of transgenic tobacco leaves was put into Mes-KCl buffer and incubated in the light intensity of 240 μmol m^-2^ s^-1^ for 2 h. And then the pre-prepared epidermal strips were transferred into another Mes-KCl buffer containing 0.5 mg mL^-1^ ALA under the same light condition for 1 h. The treated strips were sampled to observe the stomatal apertures. Meanwhile, the epidermal strips were immediately frozen with liquid nitrogen for the following experiment and stored at the -80°C refrigerator.

### Measurement of the stomatal aperture

The stomatal pictures of different genotypic tobaccos were taken by a stereoscopic fluorescence microscope (DM6B, Leica) to measure the stomatal aperture with Adobe Photoshop 6.0 (Adobe Systems, CA, USA). Forty stomata were randomly selected at each sampling time point for statistical analysis. Data presented are means ± standard error (SE).

### Determination of photosynthetic ability of transgenic tobacco plants

At least three independent transgenic lines grown 45 days were selected from T_2_ generation tobacco lines for determination of photosynthetic parameters. In the laboratory, a portable photosynthesis system analyzer (Li-6800, Lincoln, NE, USA) equipped with a leaf chamber was used to determine the gas exchange parameters of different genotypic tobacco leaves. The ambient conditions of leaf chamber were as follows: gas flow rate 500 μmol s^-1^, air chamber pressure 0.1 kPa, air humidity 60%, CO_2_ concentration 400 μmol·mol^-1^, fan speed 10000 rpm, blade temperature 27.0°C, and leaf chamber light intensity 1500 μmol m^-1^ s^-1^. After the environmental conditions of the leaf chamber were set, the net photosynthetic rate (*P*_n_, μmol m^-2^ s^-1^), intercellular CO_2_ concentration (*C*_i_, μmol mol^-1^), transpiration rate (*E*, mmol m^-2^ s^-1^) and stomata conductance (*g*_s_, mmol m^-2^ s^-1^) were recorded with 10-15 biological repetitions.

### Determination of endogenous ALA content in tobacco leaves

In order to evaluate the effects of gene transformation or exogenous ALA treatment on the endogenous ALA content, different genotypic tobaccos grown for about 4 weeks were sprayed with 0.5 mg L^-1^ ALA on the leaf surface. Forty-eight hours later, the epidermis of leaves was torn off and washed clear for endogenous ALA measurement (Zhang et al., 2011).

### Comparison of drought sensitivity of the transgenic tobacco plants

T_2_ generation seedlings of different transgenic tobaccos were treated by 15% polyethylene glycol 6000 (PEG) solution for 12 days, cultured in the growth chamber without irrigation. Then the plant growth status was compared for their drought tolerance. In biochemical aspects, we used catalase (CAT) activity assay kits (Beijing Solarbio Science & Technology Co., Lt Item No. BC0205-100T), peroxidase (POD) activity assay kits (Beijing Solarbio Science & Technology Co., Lt Item No. BC0095-100T), superoxide dismutase (SOD) (Beijing Solarbio Science & Technology Co., Lt Item No. BC0175-100T) activity assay kits, proline (PRO) content detection kits (Beijing Solarbio Science & Technology Co., Lt Item No. BC0295-100T), and malondialdehyde (MDA) content detection kits (Beijing Solarbio Science & Technology Co., Lt Item No. BC0025-100T) to test above indicators for the drought sensitivity. Each step of measurements was strictly according to the kit instructions. Three biological repetitions were conducted.

### PP2A and SnRK2.6 activity assays

The epidermal strips of 45-days-old different genotypic tobacco leaves treated with 0.5 mg L^-1^ ALA or not were ground to powder, then, total proteins were extracted using a plant protein extraction kit (Cat. No. CW0885S, Kangwei Century, China), and a BCA protein quantitative kit (Cat. No. CW0014S Kangwei Century, China) was used to determine the extracted protein content. All extracted proteins were diluted to the same concentration. Then, a plant protein phosphatase 2A (PP2A) enzyme-linked immunoassay kit (Shanghai Fusheng Industrial Co., Ltd., Item No. A024850-96T) and a SnRK2.6 enzyme-linked immunoassay kit (Item No. A089115-96T) were respectively used to detect PP2A and SnRK2.6 activity according to the manufacturer instructions (**Appendix 1**, **2**). The purified plant PP2A antibody with HRP (Horseradish Peroxidase) labeled was used to coat microtiter plate wells to make solid-phase antibody. When PP2A was added to the wells, it combined PP2A antibody labeled Horseradish peroxidase (HRP), forming antibody-antigen-enzyme-antibody complex. The 3, 3, 5, 5, -tetramethyl-benzidine (TMB) was used as the substrate, which turned blue color catalyzed by the HRP enzyme. The reaction was terminated by the addition of sulphuric acid and the color change was measured spectrophotometrically at a wavelength of 450 nm. In addition, a chelator of divalent ions (EDTA) was included in the protein phosphatase reaction buffer [50 mM Tris-HCl, pH 7, 0.1 mM Na_2_EDTA, 5 mM DTT, and 0.01% (w/v) Brij 35 (dodecyl polyglycol ether)] to inhibit the activities of PP2C and other divalent-dependent phosphatases. Protein Phosphatase Inhibitor 2 was also supplemented in the reaction buffer to specifically inhibit the activity of PP1. Three independent repeated experiments were conducted.

### Measurement of endogenous Ca^2+^, H_2_O_2_ and flavonols content of guard cells

The intracellular Ca^2+^ levels in the guard cells were determined using the fluorescent dye Fluo-3 AM (Dojindo, Japan), as described by An et al. (2016b). In turn, the fluorescent indicator H_2_DCF-DA as described by An et al, 2016a; An et al, 2016b was applied to test the endogenous H_2_O_2_ levels, while endogenous flavonols were measured with the fluorescent indicator dye diphenylboric acid 2-aminoethyl ester (DPBA), as described by Watkins et al. (2014).

### Yeast two-hybrid

The full length of *MdPP2AC* was ligated to the pGBKT7 vector (Clontech) as bait. The full length of *MdPTPA* and *MdSnRK2.6* were ligated to pGADT7 vector as the prey, respectively. In addition, *MdSnRK2.6* was ligated to the pGBKT7 vector (Clontech) as bait. The full length of *MdPTPA* was ligated to pGADT7 vector as the prey. Plasmids of the prey and bait were mated and transformed into the yeast strain Y2HGold (Clontech, USA), and the mating cultures were spread on stringent selective medium plates containing X-α-gal (20 mg mL^-1^) and 1 mg mL^-1^ Aureobasidin A (AbA). The plates were incubated at 30°C for 3 to 5 d and checked every 12 h for the development of blue color. Plasmids pGBKT7-Lam with pGADT7-T control vector and pGBKT7-*MdPP2AC* with pGADT7 were used as negative controls, and the pGBKT7-53 and pGADT7-T vectors were used as the positive controls. The primer sequences used are listed in **Table S1**.

### Yeast three-hybrid and β-Galactosidase activity assay

Firstly, the CDS sequence of *MdPTPA* was ligated to the pBridge vector via single NotI site and then the CDS sequence of *MdPP2AC* was ligated into the pBridge vector (Clontech) using EcoRI and BamHI sites, constructing the recombination plasmids pBridge-*MdPP2AC*-*MdPTPA*. The full length of *MdSnRK2.6* was ligated to pGADT7 vector via EcoRI and BamHI sites. Plasmids of pBridge-*MdPP2AC-MdPTPA* and pGADT7-*MdSnRK2.6* were mated and transformed into the yeast strain AH109 (Clontech, USA), and the mating cultures were spread on stringent selective medium plates SD/-Leu/-Trp, SD/-Ade/-His/-Leu/-Trp, SD/-Ade/-His/-Leu/-Trp/-Met, respectively. The *MdPTPA* was cloned into MCS II of pBridge vector which was located downstream of an HA epitope and a second NLS, to form a bridge protein. The resulting fusion protein was conditionally expressed from the *Met25* promoter in response to methionine levels in the medium (**Appendix 3**). The expression of fusion protein was suppressed in the presence of 1 mM methionine and expressed in the absence of methionine. The plates were incubated at 30°C for 3 to 5 d and checked every 12 h for the development of yeast monoclonal. Subsequently, the β-galactosidase assay kit (Shanghai Fusheng Industrial Co., Ltd., Item No. A087910-96T) was used to detect the β-galactosidase activity. The primer sequences used are listed in **Table S1**.

### Statistics and reproducibility

The data are means of at least three independent biological replications. Statistical analyses were performed a two-sided Student’s t-test or a one-way ANOVA followed by mean separation with Tukey’s honestly significant difference test or Duncan’s multiple range test.

## Results

### Overexpression of *MdPTPA*, *MdPP2AC* or *MdSnRK2.6* affects stomatal movement in transgenic tobaccos

**Figure 1:**
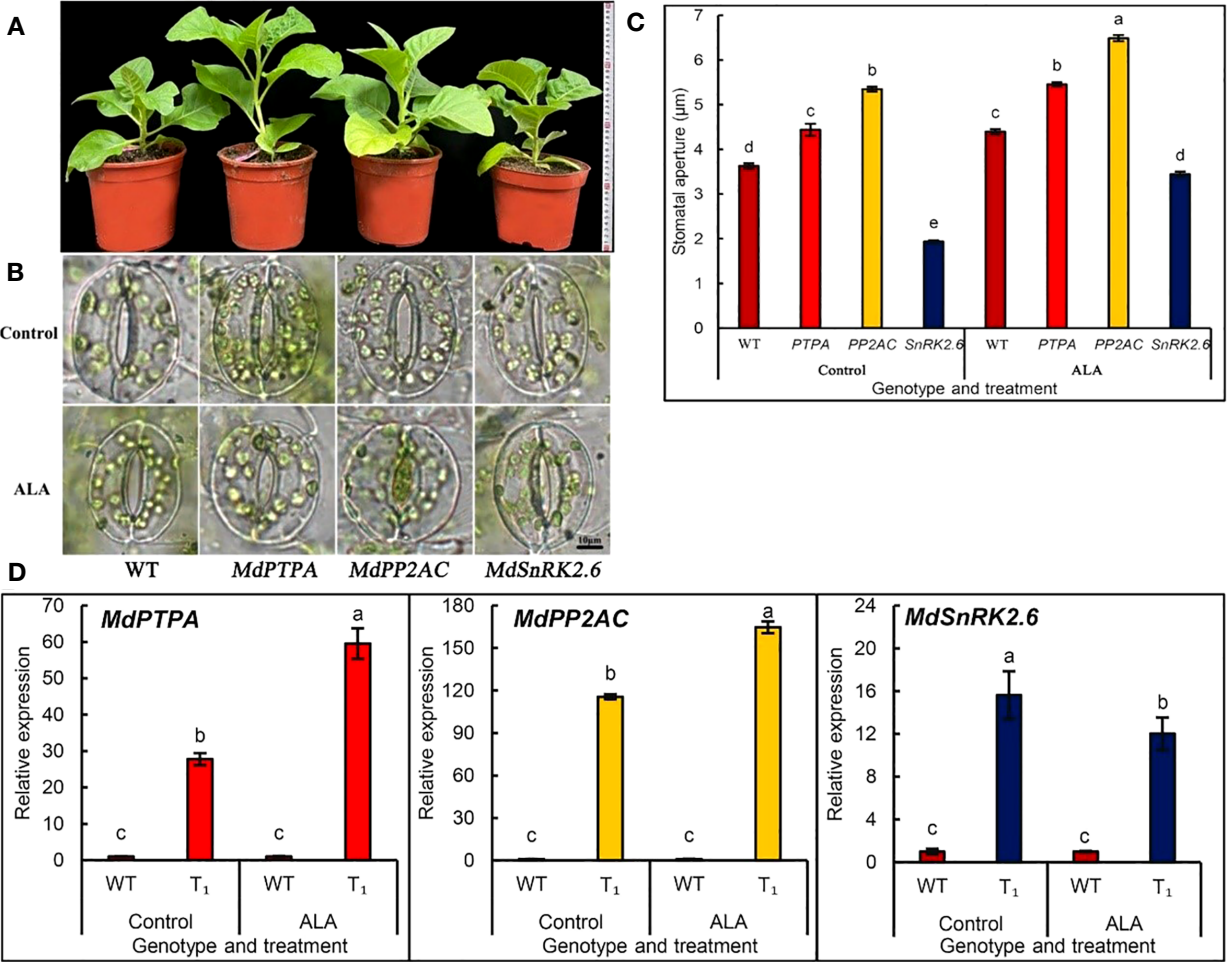
The transgenic plants which overexpressed *MdPTPA*, *MdPP2AC* and *MdSnRK2.6*, respectively, in tobacco. Genetic transformation was carried out by the leaf disc method with *A. tumefaciens* GV3101 as the vector to obtain stable transgenic plants. **(A)** the potted cultured plants. **(B)** the stomatal photos of different genotypes treated with or without 0.5 mg L^-1^ ALA. Scale bar = 10 µm. **(C)** the stomatal apertures of different genotypes of tobacco treated with or without 0.5 mg L^-1^ ALA. **(D)** RT-qPCR detection of *MdPTPA*, *MdPP2AC* and *MdSnRK2.6* expressions in the transgenic tobacco plants, respectively, treated with ALA or not. During the experiments, the isolated epidermal strips of wild-type and transgenic tobacco were incubated at 25°C in MES-KCl buffer without plant hormones (Control). After 2 h illumination pretreatment (240 µmol m^−2^ s^−1^), the strips were transferred into the same buffer but containing 0.5 mg L^−1^ ALA and illuminated one hour more, then the RNA was extracted for RT-qPCR. The values are the means ± SE of three biological replicates and the different letters represent significant difference at *P* = 0.05.

We established different transgenic tobacco plants which over-expressed *MdPTPA*, *MdPP2AC* or *MdSnRK2.6* of apple. We confirmed the gene transformation by PCR, GUS (**Figure S1**) and RT-PCR (**Figure 1D**). Only the homozygous positive transgenic lines were used in the following experiments. From **Figure 1A**, the plants of OE-*MdPTPA* or OE-*PP2AC* grew much better than that of the wild type (WT), as well as OE-*SnRK2.6*. These suggest that overexpression of *MdPTPA* or *MdPP2AC* may promote plant growth under normal conditions. When the stomatal aperture was measured, the results showed that OE-*MdPTPA* or OE-*MdPP2AC* promoted stomatal aperture, while OE-*MdSnRK2.6* induced stomatal closure (**Figures 1B, C**). When the leaf epidermis was treated by exogenous ALA, all stomatal apertures were promoted, suggesting that no matter which gene was over-expressed in tobaccos, ALA promoted all their stomatal opening. Therefore, ALA may act at the upper stream to regulate all the gene expressions in tobacco. Additionally, we detected the corresponding gene expressions of tobacco and the results showed that ALA significantly promoted expressions of *NtPTPA* and *NtPP2AC* but inhibited the *NtSnRK2.6* (**Figure S2**). It is consistent with the heterogenous expressions of *MdPTPA*, *MdPP2AC* and *MdSnRK2.6* in tobaccos (**Figure 1D**).

### Overexpression of *MdPTPA*, *MdPP2AC* or *MdSnRK2.6* affects PP2A and SnRK2.6 activities in transgenic tobaccos

**Figure 2:**
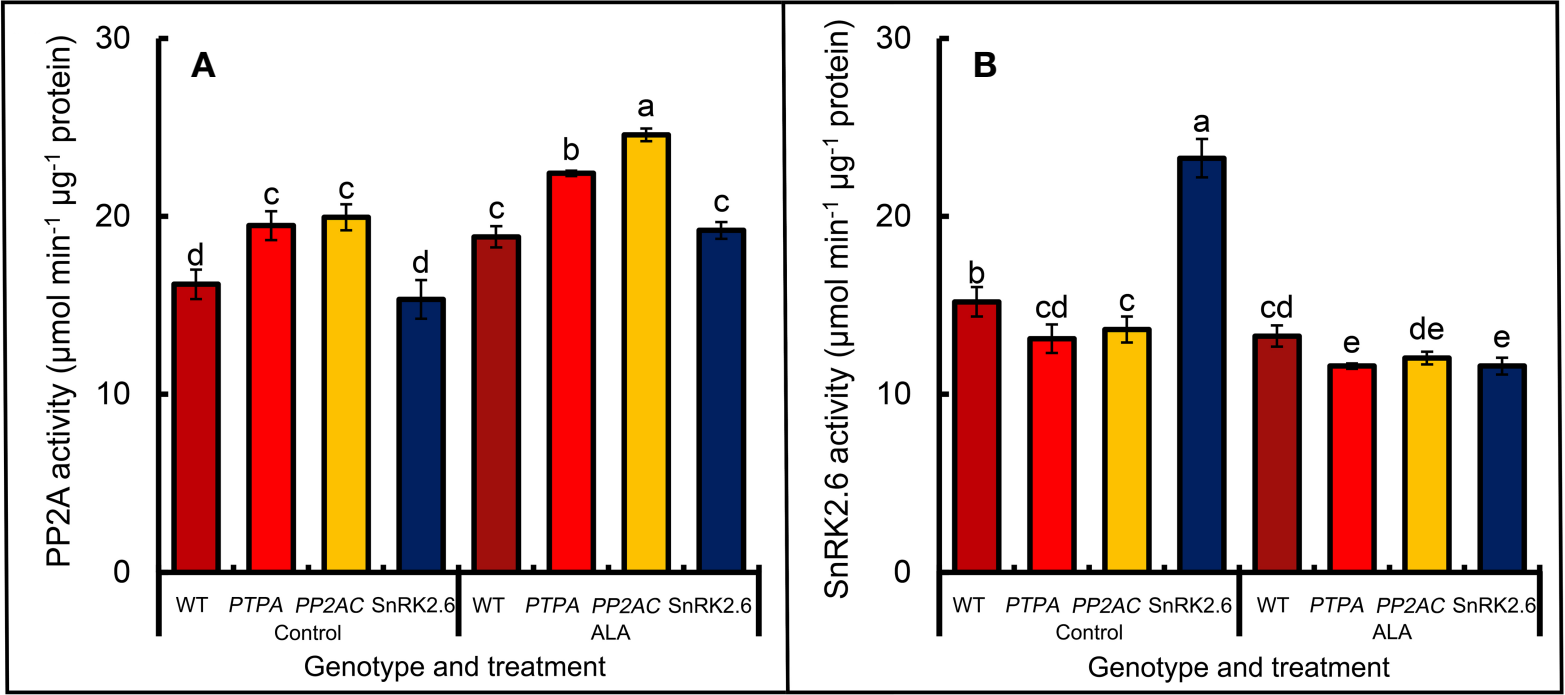
Effects of ALA on the PP2A **(A)** and SnRK2.6 **(B)** activities in the leaf epidermis of different genotypes of tobaccos. The isolated epidermal strips of 45-day-old tobaccos were incubated at 25°C in MES-KCl buffer with or without exogenous 0.5 mg L^−1^ ALA under light (240 µmol m^−2^ s^−1^) for 1 (h) The values are the means ± SE from three biological replicates. The same letters above the bars in each enzyme indicate no significant differences at *P* = 0.05 level.

**Figure 2A** shows that over-expression of *MdPTPA* or *MdPP2AC* significantly increased PP2A activity in transgenic tobacco leaves, while OE-*MdSnRK2.6* had no effect on the PP2A activity. If the epidermis was treated by exogenous ALA, the PP2A activity was promoted in all genotypic including the WT plants. From **Figure 2B**, over-expression of *MdPTPA* or *MdPP2AC* depressed the SnRK2.6 activities in transgenic tobacco leaves, while exogenous ALA treatment aggravated the effect. Conversely, over-expression of *MdSnRK2.6* significantly increased SnRK2.6 activity in tobacco leaves, while exogenous ALA treatment fully eliminated the effect. These data suggest that PTPA and PP2AC must work at the upper stream of the ALA signal route to negatively regulate SnRK2.6 activity, but OE-*SnRK2.6* has no effect on PP2A activity.

### Over-expression of *MdPTPA*, *MdPP2AC* or *MdSnRK2.6* affects Ca^2+^, H_2_O_2_ and flavonol levels in the guard cells in the transgenic tobaccos

**Figure 3:**
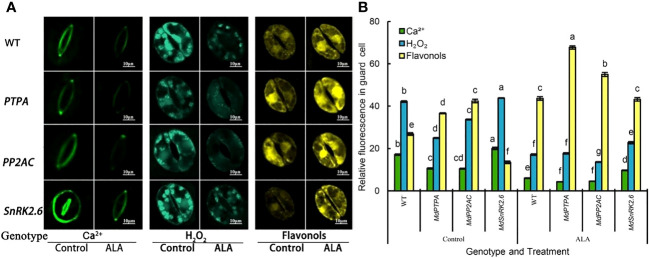
Effects of ALA treatment on the Ca^2+^, H_2_O_2_ and flavonol content in the stomatal guard cells of different genotypes tobacco, where *MdPTPA*, *MdPP2AC* and *MdSnRK2.6* are the over-expressing transgenic plants. **(A)** The isolated epidermal strips of 45-days-old tobacco were incubated at 25°C in MES-KCl buffer with or without exogenous 0.5 mg L^−1^ ALA under light (240 µmol m^−2^ s^−1^) for 1 h, then loaded with 1 μmol L^-1^ fluorescence probe fluo-3 AM incubated in the dark at 4 °C for 2 h, 50 µM H_2_DCF-DA for 30 min in darkness at 25 °C, and 2.52 mg mL^−1^ diphenylboric acid 2-aminoethyl ester (DPBA), respectively, for 30 min in darkness and the fluorescence intensity was detected by a super resolution laser confocal microscope (LSM800, ZEISS, Germany). Excitation light (ex) = 488 nm, emitted light (em) = 525 ± 15 nm, power 50%, zoom 16, mild scanning, frame 512 × 512. For each treatment, fluo-3 AM bound to Ca^2+^ showed green fluorescence, H_2_DCF-DA bound to H_2_O_2_ produced blue green fluorescence and DPBA bound to flavonols issued yellow fluorescence. Scale bar: 10 µm. **(B)** The relative fluorescence intensity of Fluo-3 AM, H_2_DCF-DA and DPBA in guard cells of each treatment, which was measured by Image J software. The values are the means ± SE of 15 replicate measurements. The same letters above the bars in each item indicate no significant difference at *P* = 0.05 level.

Since stomatal aperture is associated with the content of Ca^2+^, H_2_O_2_, and flavonols in the guard cells, we detected their levels in the transgenic tobaccos. It is obvious that the Ca^2+^ and H_2_O_2_ content in the guard cells of OE-*MdPTPA* and OE-*MdPP2AC* transgenic plants were significantly down-regulated, while the flavonol content was obviously up-regulated. Conversely, the guard cells of OE-*MdSnRK2.6* contained more Ca^2+^ and H_2_O_2_ with less flavonols than the wild type (**Figure 3**). These suggest that OE-*MdPTPA* and OE-*MdPP2AC* decreased Ca^2+^ and H_2_O_2_ levels with more flavonol accumulation in the guard cells. On the other hand, OE-*MdSnRK2.6* increased Ca^2+^ and H_2_O_2_ levels with less flavonols in the guard cells. Furthermore, ALA promoted flavonol accumulation and depressed the Ca^2+^ and H_2_O_2_ content in all transgenic plants, suggesting that ALA’s functions can cover all these genes.

### Overexpression of *MdPTPA*, *MdPP2AC* or *MdSnRK2.6* affects photosynthetic gas exchanges in tobaccos

**Figure 4:**
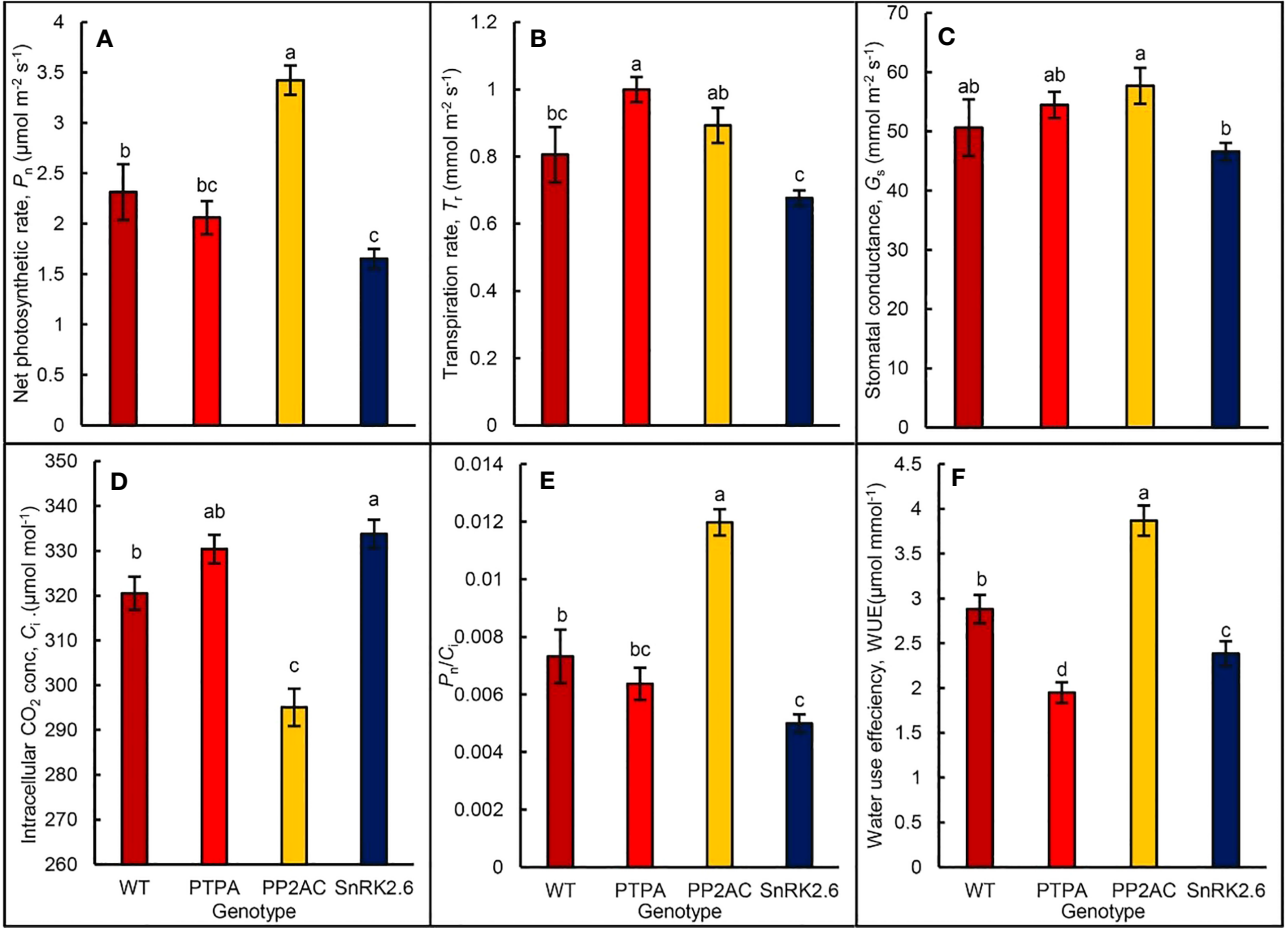
The gas exchange parameters of different genotypic tobaccos, where *MdPTPA*, *MdPP2AC* and *MdSnRK2.6* were the over-expressing transgenic plants. A portable photosynthesis system (LI-6800, Li-Cor) was used to measure the parameters in the leaves of 45-day old plants. **(A)** the net photosynthetic rate (*P*_n_); **(B)** transpiration rate (*T*_r_), **(C)** stomatal conductance (*G*_s_); **(D)** intercellular CO_2_ concentration (*C*_i_); **(E)** instantaneous carboxylation efficiency (*P*_n_/*C*_i_); **(F)** water use efficiency (WUE). The values are the means ± SE of 10 replicate measurements. The same letters above the bars in each item indicate no significant difference at *P* = 0.05 level.

When the leaf photosynthetic gas exchanges of the different genotypic tobaccos were measured by a portable photosynthesis system, the results showed that net photosynthetic rate (*P*_n_) was significantly promoted in the OE-*MdPP2AC* but inhibited in the OE-*MdSnRK2.6*, which was not significantly affected by OE-*MdPTPA* when compared with the WT (**Figure 4A**). The *P*_n_ in OE-*MdPP2AC* and OE-*MdSnRK2.6* was 148.28% and 74.17% of the WT, suggesting that the former gene promoted leaf photosynthesis while the latter inhibited photosynthesis. Furthermore, OE-*MdPTPA* or OE-*MdPP2AC* tended to increase leaf transpiration (*T*_r_) and stomatal conductance (*G*_s_), while OE-*MdSnRK2.6* tended to decrease *T*_r_ and *G*_s_ of the transgenic tobacco. If comparisons were between OE-*MdSnRK2.6* and OE-*MdPTPA* especially OE-*MdPP2AC*, the differences were significant (**Figures 4B, C**). These suggest that up-regulation of signals in the ALA route such as *PTPA* or *PP2AC* will increase stomatal opening and leaf transpiration. If the signal in the ABA route such as *SnRK2.6* was up-regulated, *G*_s_ and *T*_r_ were significantly depressed. It is interesting to notice that the intercellular CO_2_ concentration (*C*_i_) in the OE-*MdPP2AC* tobacco leaves was significantly decreased, while increased in the OE-*MdSnRK2.6* compared with the WT (**Figure 4D**). It may suggest that both gene transformations affect not only stomatal movement but also CO_2_ fixation. Therefore, the instantaneous carboxylation efficiency (*P*_n_/*C*_i_) in the OE-*MdPP2AC* transgenic tobacco was increased while decreased in the OE-*SnRK2.6* (**Figure 4E**). Similarly, the water use efficiencies (WUE) were significantly decreased in the OE-*MdPTPA* and OE-*SnRK2.6* while increased in the OE-*MdPP2AC*, compared to the WT (**Figure 4F**). The lowest WUE in the OE-*MdPTPA* implies that over-expression of the gene may promote stomatal aperture without any promotive effect on photosynthesis. In addition, we detected the endogenous ALA content of OE-*MdPTPA*, OE-*MdPP2AC* or OE-*MdSnRK2.6* tobaccos, including treated with exogenous ALA. The results showed that there was no significant difference in the endogenous ALA content among different genotypic tobaccos regardless of ALA treatment (**Figure S3**), suggesting that the gene transformations had no effect on the endogenous ALA. Therefore, ALA is at the upstream signal of *MdPTPA*, *MdPP2AC* or *MdSnRK2.6*.

### Overexpression of *MdPTPA*, *MdPP2AC* or *MdSnRK2.6* affects drought tolerance in transgenic tobaccos

**Figure 5:**
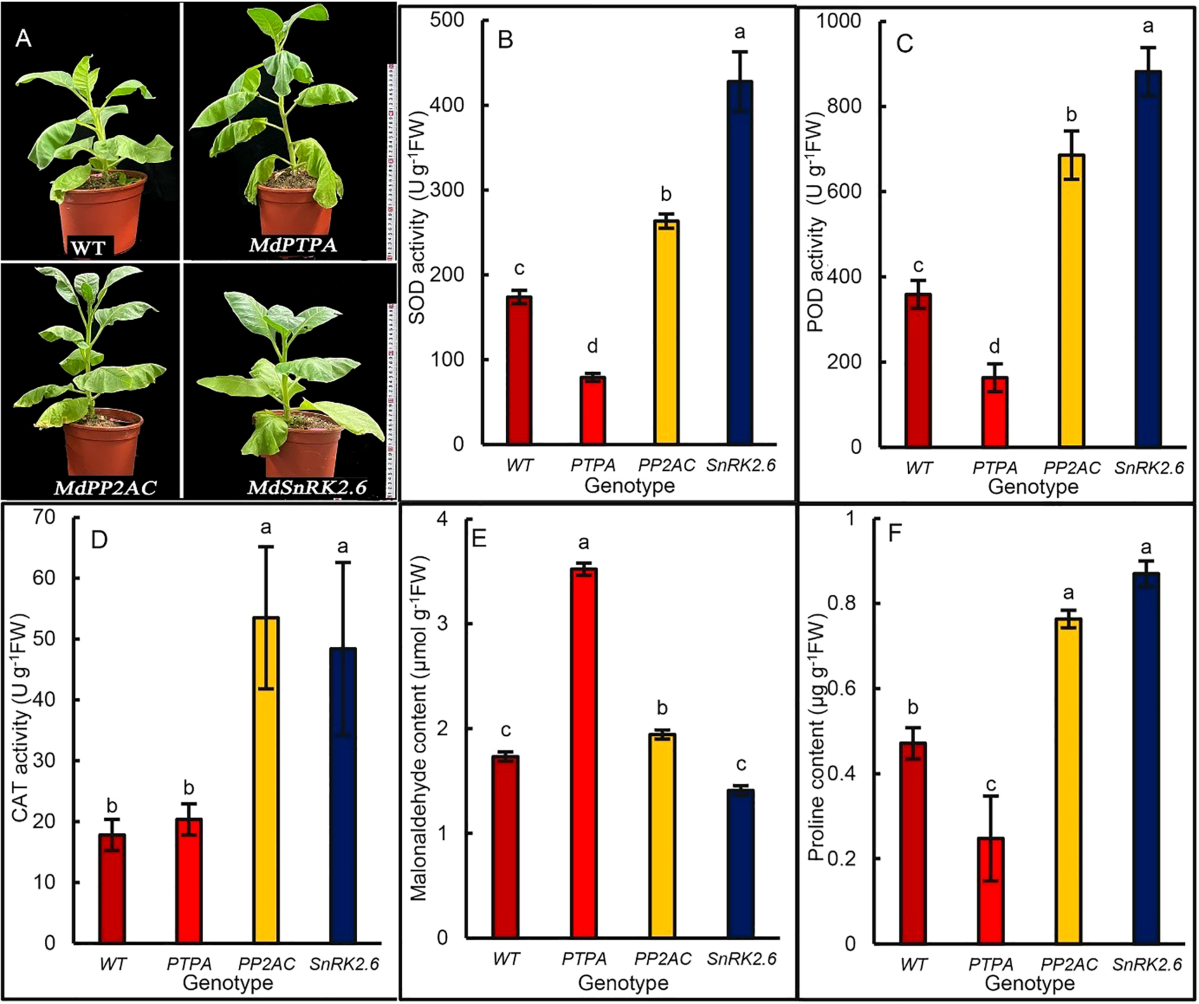
Plant appearance and biochemical differences among genotypic tobaccos after osmotic stress. The 45-day old potted tobacco plants were treated with 15% polyethylene glycol 6000 for 12 d, and the antioxidant enzyme activities and proline content were measured. **(A)** Growth phenotype of different genotypes of tobacco plants, where *MdPTPA*, *MdPP2AC* and *MdSnRK2.6* were the transgenic tobacco, respectively. The *MdPTPA* transgenic tobacco wilted more severely than *MdPP2AC* and *MdSnRK2.6* plants. **(B–D)** the activity of superoxide dismutase (SOD), peroxidase (POD) and catalase (CAT), respectively; **(E)** the content of malondialdehyde (MDA), **(F)** the proline content. The values are the means ± SE from three biological replicates. The same letters indicate no significant differences at *P* = 0.05 level.

To explore the effects of gene transformation on drought tolerance of the transgenic tobaccos, we treated the plants with 15% PEG for 12 days. The results showed that the leaves of OE-*MdPTPA* wilted severely, while OE-*MdPP2AC* and OE-*MdSnRK2.6* kept rather steady (**Figure 5A**). These may suggest that *MdPTPA* negatively while *MdPP2AC* and *MdSnRK2.6* positively regulated the drought tolerance of plants. From **Figures 5B–D**, the activities of superoxide dismutase (SOD), peroxidase (POD) and catalase (CAT) in the OE-*MdPP2AC* and OE-*MdSnRK2.6* tobaccos were much higher, while that in the OE-*MdPTPA* were not as high as, even lower than that of the WT. So was the proline content (**Figure 5F**). Conversely, the malonaldehyde content (MDA) in the OE-*MdPTPA* was the highest among the genotypes, while that in the OE-*MdPP2AC* was only slightly higher than that of the WT. Furthermore, OE-*MdSnRK2.6* inhibited the MDA accumulation under stressful condition (**Figure 5E**). These data suggest that the drought tolerance of tobacco was promoted by OE-*MdSnRK2.6* and OE-*MdPP2AC* but impaired by OE-*MdPTPA.*

### MdPTPA promotes the interactions between *MdPP2AC* and *MdSnRK2.6*

**Figure 6:**
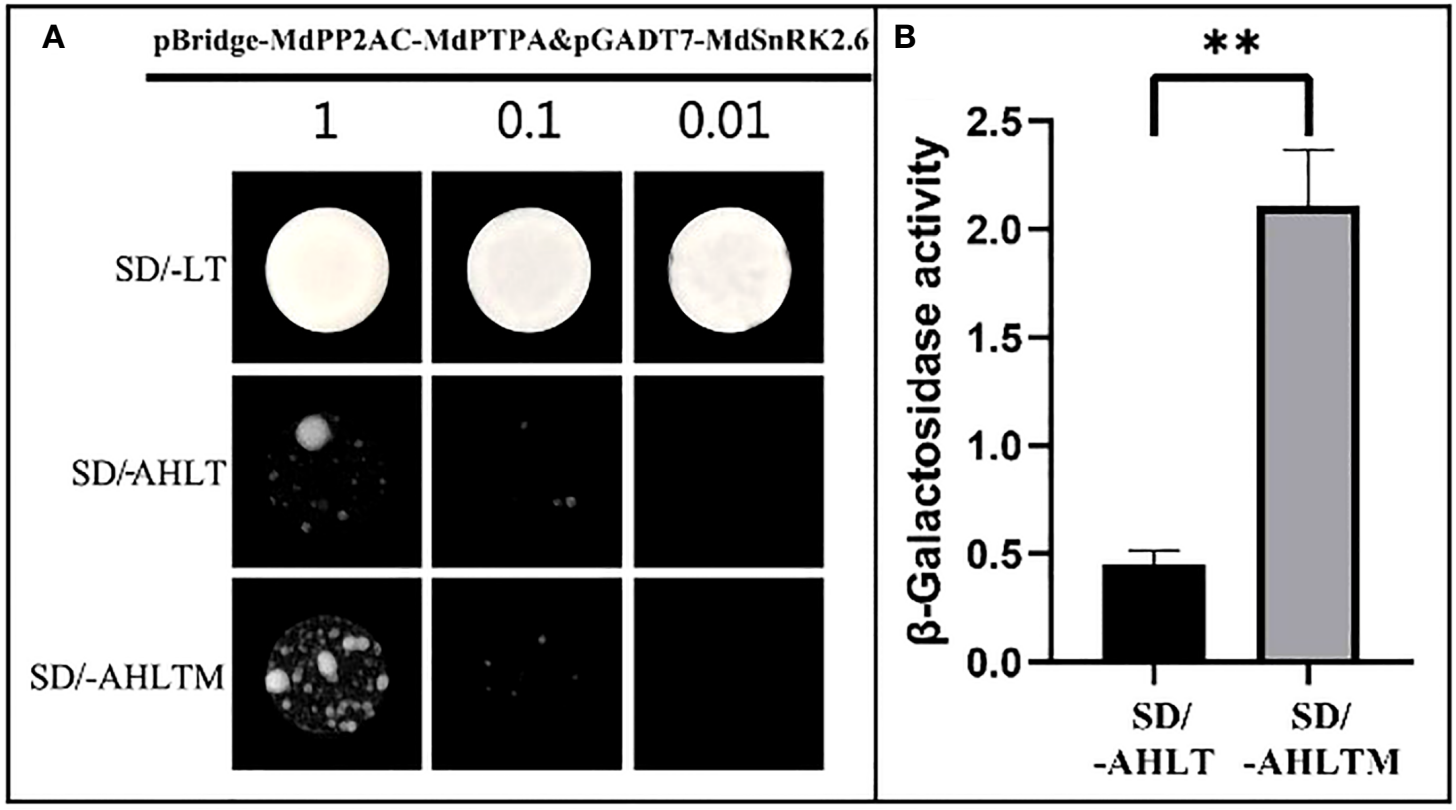
MdPTPA promoting the interaction of MdPP2AC with MdSnRK2.6 demonstrated by Yeast three-hybrid. **(A)** Yeast three-hybrid (Y3H) assay, which shows that MdPTPA promotes the interactions between MdPP2AC and MdSnRK2.6. SD/-LT: SD/-Leu/-Trp, SD/-AHLT: SD/-Ade/-His/-Leu/-Trp, SD/-AHLT/M: SD/-Ade/-His/-Leu/-Trp/Met. Three independent repeated experiments were performed with similar results. **(B)** β-galactosidase activity assay. The data are the means ± SE of three biological replicates and the symbol ** represents the difference significant at *p* = 0.01.

The MdPP2AC has been shown to interact with MdPTPA and MdSnRK2.6, respectively (**Figure S4**), however, no interaction was found between MdPTPA and MdSnRK2.6 (**Figure S5**). Then, whether MdPTPA is involved in the interaction MdPP2AC with MdSnRK2.6 was not known. Therefore, the yeast three-hybrid technology was employed. The results showed that yeast monoclonals grew well and consistently, even if after 100-fold dilution, in the SD/-LT medium. When yeast was cultured in the SD/-AHLT medium, the monoclonals still grew although weakly, indicating that MdPP2AC interacted with MdSnRK2.6. When the yeast monoclonals were cultured on the SD/-AHLTM medium, where methionine was absent and then *MdPTPA* was induced to express, the yeast monoclonals grew better than that on SD/-AHLT (**Figure 6A**). The enzyme assay also showed that the β-galactosidase activity of yeast monoclonals on SD/-AHLTM medium was 4.69 times as high as that on SD/-AHLT (**Figure 6B**). These results suggest that MdPTPA promoted the interaction of MdPP2AC with MdSnRK2.6, and the yeast grew better with higher enzyme activity.

## Discussion

Since stomatal closure can reduce leaf transpiration and water dissipation, which is significant for water-saving agricultural production, most of studies on the regulatory mechanisms of stomatal movement mainly focus on the stomatal closure (Dodd, 2003; Acharya and Assmann, 2009). But in fact, stomatal opening also has important scientific and production significance. The increase of stomatal opening is conducive to CO_2_ entry into mesophyll cells, which is necessary for leaf photosynthesis and plant growth. Today, the international community all concern “the Double Carbon” (Pan, 2021), namely carbon peak and carbon neutrality (CPCN). Different countries have introduced their own CPCN goals and policies (Wang et al., 2021). Green plants capture atmospheric CO_2_ through open stomata and convert it to carbohydrates through photosynthesis and produce human food or processing raw materials. The annual biomass energy potential in China has been estimated about 460 million tons of standard coal (Yao, 2021). Therefore, the biological negative carbon emission is very important in ecological equilibrium. In present study, we found that transformation of *MdPTPA* or *MdPP2AC* of apple into tobacco significantly up-regulated the gene expressions and PP2A activity, which further promoted the leaf stomatal aperture and up-regulated by exogenous ALA (**Figures 1**, **2**). Thus, *MdPTPA/MdPP2AC* may function at the upstream of PP2A signaling route, promoting stomatal opening regulated by ALA. It is consistent with the results from detached apple leaves (Chen and Wang, 2022; Chen et al., 2023). From **Figure 1A**, we observed that the growth of OE-*MdPTPA*/OE-*MdPP2AC* was better than the WT, suggesting that the two genes are beneficial for plant growth. From **Figure 4A**, the *P*_n_ of OE-*MdPP2AC* was 48.28% higher than that of the WT, suggesting that the gene may have great potential for plants to convert more atmospheric CO_2_ into organic compounds.

SnRK2.6 is a key regulator of ABA signaling route to induce stomatal closure (Daszkowska-Golec & Szarejko, 2013). Our previous study has revealed that it is the crossing point for ALA and ABA signaling routes (Chen et al., 2023). In the detached apple leaves, ALA induces PP2AC phosphorylation and the holoenzyme activity. Then, the increased PP2A activity can induce SnRK2.6 dephosphorylation and block ABA signaling transduction downward. When ABA is present, ABA signals induce SnRK2.6 phosphorylation, then the downstream signal components, such as H_2_O_2_ and Ca^2+^ levels in the guard cells are upregulated with less flavonol accumulation, which induces stomatal closure. Conversely, when ALA is added, *PTPA* and *PP2AC* gene expressions are upregulated, which induces PP2A activity increase, downregulating *SnRK2.6* expression and dephosphorylating SnRK2.6 protein and decreasing SnRK2.6 activity in apple epidermis (Chen et al., 2023). In the current study, we observed a similar regulatory mechanism with the transgenic tobaccos. When *MdSnRK2.6* was transformed into tobacco, the stomatal aperture was significantly decreased compared to the WT. Furthermore, exogenous ALA down-regulated the *SnRK2.6* expression (**Figure 1D**) and SnRK2.6 enzyme activity (**Figure 2B**), which caused stomatal aperture greater than the control (**Figures 1B, C**). All the regulatory effects are similar with the detached apple leaves (Chen et al., 2023). On the other hand, Yu et al (2015) proposed that AtPP2A dephosphorylated ABI5, a member of the subfamily of bZIP transcription factors, to stop ABA signaling. Whether similar functions of MdPP2A occur in ALA-ABA regulating stomatal movement needs study further.

Ca^2+^ and H_2_O_2_ are involved in stomatal regulation (Lefoulon et al., 2016; Hauser et al., 2017; Mathe et al., 2019), which has been found in ALA-ABA regulated stomatal movement in apple (Chen et al., 2014a) and *Arabidopsis* (An et al., 2016b). Furthermore, ALA-promoted stomatal opening depends on flavonol accumulation in guard cells, especially in cell nuclei (Liu et al., 2016). In present work, we found that OE-*MdPTPA* or OE-*MdPP2AC* significantly increased the flavonol content while depressed Ca^2+^ and H_2_O_2_ in the guard cells in the transgenic tobaccos. Yet, the situations in the OE-*MdSnRK2.6* were opposite. Exogenous ALA up-regulated the positive factors for stomatal opening but down-regulated the negative factors in all genotype plants (**Figure 3**). Thus, we provide genetic evidence that Ca^2+^, H_2_O_2_ and flavonol levels act at the down-stream of PTPA, PP2AC and SnRK2.6 signaling route during ALA-induced stomatal opening.

It is well known that when plants are stressed by drought or salinity, endogenous ABA is often accumulated greatly, which leads to stomatal close and water loss depression (Zhang et al., 2006; Xu et al., 2021). It has been considered as one of the most important mechanisms for plants to adapt the severe environment. However, the accumulated ABA tends to depress photosynthesis (Šantrůček et al., 2003; Huntingford et al., 2015). ALA can promote plant drought tolerance (Al-Khateeb, 2006; Li et al., 2011; Kosar et al., 2015) although it causes higher stomatal conductance (Liu et al., 2013). It seems conflicting with the existing theories, because the larger stomatal aperture, the more water loss, and the more severely plants wilt under drought condition. Recently, Cai et al. (2020) proposed a new hypothesis to explain the paradox. They found that exogenous ALA-treated plants had greater ability to absorb water from a high osmotic substrate than those without ALA treatment, which was transported to the aboveground by ALA-upregulated aquaporins. Therefore, even the stomatal aperture and transpiration were higher, the water homeostasis of whole plants was kept well. Furthermore, ALA promoted leaf net photosynthesis and water use efficiency. In present work, we also found that OE-*MdPP2AC* improved stomatal opening and *P*_n_, as well as drought tolerance (**Figure 5**), while OE-*MdSnRK2.6* induced drought tolerance but depressed leaf *P*_n_ (**Figure 4**). Thus, OE-*MdPP2AC* and OE-*MdSnRK2.6* are beneficial for drought tolerance of plants. Furthermore, the antioxidant enzyme activities including SOD, POD, and CAT in the OE-*MdPP2AC/MdSnRK2.6* transgenic tobaccos were all promoted with lower MDA accumulation, compared with the WT and OE-*MdPTPA* (**Figure 5**). Proline, an important osmotic solute was accumulated in the two transgenic plants but not in the OE-*MdPTPA*, suggesting that *MdPTPA* can promote stomatal open but impair drought tolerance. Therefore, we propose that ALA is a non-protein amino acid, which not only induces stomatal opening mediated by PP2AC to block SnRK2.6 signaling from ABA, but also promotes antioxidant enzyme activities, osmotic adjustment, and stress tolerance, the latter is often considered to be the functions of ABA. Growth or survival is a contradiction for plants when stress is coming. ABA seems to improve survival rather than growth, while ALA can improve both growth and survival for plants (An et al., 2016a; Cai et al., 2020). SnRK2.6, the key component of ABA signaling route, confers plant stress tolerance but not growth; PTPA, a positive activator of PP2A, can promote PP2A activity, stomatal aperture, and plant growth under normal condition, but impair drought tolerance of plants. Only PP2AC, a key factor in the ALA signaling route can promote stomatal aperture, leaf photosynthesis, plant growth, and drought tolerance.

In the previous study, we used Y2H assays and found MdPP2AC interacted with MdPTPA and MdSnRK2.6, respectively (Chen et al., 2023). In current study, we confirmed the interactions (**Figure S4**). However, we did not find interaction between MdPTPA and MdSnRK2.6 (**Figure S5**). Nevertheless, we used Y3H assay and found that three of them interacted among (**Figure 6**). Therefore, we consider that MdPTPA interacts with MdPP2AC, promoting the latter interacting with MdSnRK2.6, which aggravates dephosphorylation of MdSnRK2.6 and defunction of ABA signaling.

**Figure 7:**
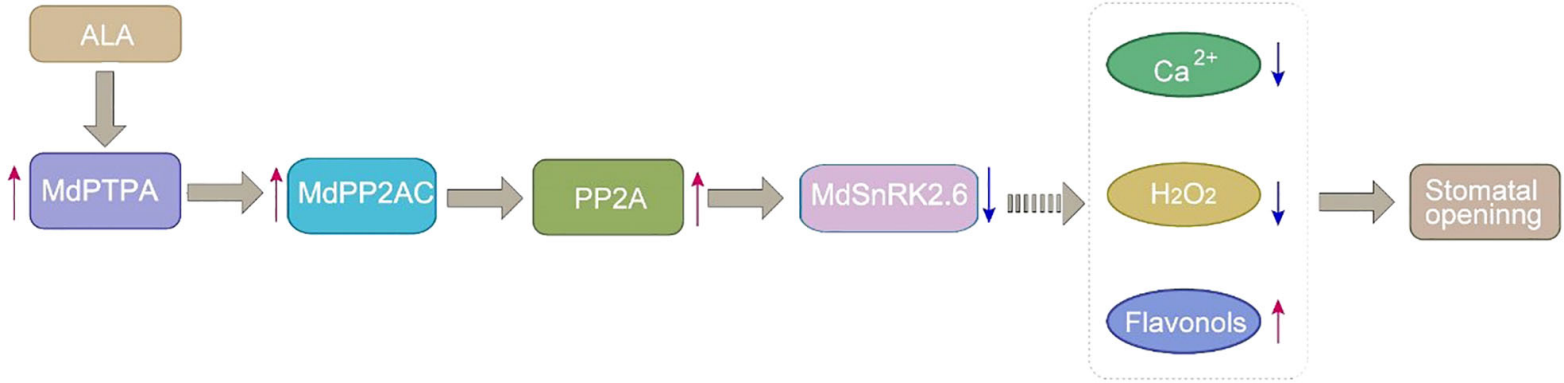
A proposed working model that ALA antagonizes ABA signaling at SnRK2.6 to promote stomatal opening. Red arrows represent positive regulation, bule arrows represent negative regulation.

Based on the findings here and the previous reports, we propose a working model for ALA to antagonize ABA signaling at SnRK2.6 to promote stomatal opening in **Figure 7**. When ALA is present, it up-regulates *MdPTPA* and *MdPP2AC* expressions, increasing PP2A activity. The phosphorylated PP2A interacts MdSnRK2.6 and dephosphorylates it. Then, the deactivated MdSnRK2.6 permits flavonol accumulation in nuclei, leading to depression of cytoplasmic Ca^2+^ and H_2_O_2_ levels. Consequently, ALA induces stomatal opening (Chen et al., 2023).

## Data availability statement

The original contributions presented in the study are included in the article/**Supplementary Material**. Further inquiries can be directed to the corresponding author.

## Author contributions

LW made the major contributions in designing the experiments and revising manuscript. ZC performed all the experiments and wrote the manuscript. JZ was responsible for the format modification of the article. All authors read and approved the manuscript.

## References

[B1] Acharya B. R. Assmann S. M. (2009). Hormone interactions in stomatal function. Plant Mol. Biol. 69, 451–462. doi: 10.1007/s11103-008-9427-0 19031047

[B2] Akram N. A. Ashraf M. (2013). Regulation in plant stress tolerance by a potential plant growth regulator, 5-aminolevulinic acid. J. Plant Growth Regul. 32, 663–679. doi: 10.1007/s00344-013-9325-9

[B3] Al-Khateeb S. A. (2006). Promotive effect of 5-aminolevulinic acid on growth, yield and gas exchange capacity of barley (*Hordeum vulgare* L.) grown under different irrigation regimes. J. King Saud. Univ. 18, 103–111. doi: 10.3923/ja.2006.45.49

[B4] An Y. Y. Feng X. X. Liu L. B. Xiong L. J. Wang L. J. (2016a). ALA-induced flavonols accumulation in guard cells is involved in scavenging H_2_O_2_ and inhibiting stomata closure in Arabidopsis cotyledons. Front. Plant Sci. 7. doi: 10.3389/fpls.2016.01713 PMC5108921 27895660

[B5] An Y. Y. Liu L. B. Chen L. H. Wang L. J. (2016b). ALA inhibits ABA-induced stomatal closure via reducing H_2_O_2_ and Ca^2+^ levels in guard cells. Front. Plant Sci. 7, 482. doi: 10.3389/fpls.2016.00482 27148309 PMC4826879

[B6] An Y. Y. Xiong L. J. Hu S. Wang L. J. (2020). PP2A and microtubules function in 5-aminolevulinic acid-mediated H_2_O_2_ signaling in *Arabidopsis* guard cells. Physiol. Plant 168, 709–724. doi: 10.1111/ppl.13016 31381165

[B7] Cai C. Y. He S. S. An Y. Y. Wang L. J. (2020). Exogenous 5-aminolevulinic acid improves strawberry tolerance to osmotic stress and its possible mechanisms. Physiol. Plant 168, 948–962. doi: 10.1111/ppl.13038 31621913

[B8] Chen J. Hu R. Zhu Y. Shen G. Zhang H. (2014a). *Arabidopsis* phosphotyrosyl phosphatase activator is essential for protein phosphatase 2A holoenzyme assembly and plays important roles in hormone signaling, salt stress response, and plant development. Plant Physiol. 166, 1519–1534. doi: 10.1104/pp.114.250563 25281708 PMC4226365

[B9] Chen L. H. Liu L. B. An Y. Y. Zhang Z. P. Wang L. J. (2014b). Preliminary studies on the possible mechanism underlying 5-aminolevulinic acid-induced stomatal opening in apple leaves. Acta Hortic. Sin. 41, 1965–1974. doi: 10.16420/j.issn.0513-353x.2014.10.002

[B10] Chen Z. Wang L. J. (2022). ALA upregulates MdPTPA expression to increase the PP2A activity and promote stomatal opening in apple leaves. Plant Sci. 325, 111490. doi: 10.1016/j.plantsci.2022.111490 36216297

[B11] Chen Z. An Y. Y. Wang L. J. (2023). ALA reverses ABA-induced stomatal closure by modulating PP2AC and SnRK2.6 activity in apple leaves. Hortic. Res. 10 (6), uhad067. doi: 10.1093/hr/uhad067 37287446 PMC10243991

[B12] Daszkowska-Golec A. Szarejko I. (2013). Open or close the gate - stomata action under the control of phytohormones in drought stress conditions. Front. Plant Sci. 4, 138. doi: 10.3389/fpls.2013.00138 23717320 PMC3652521

[B13] DeLong A. (2006). Switching the flip: protein phosphatase roles in signaling pathways. Curr. Opin. Plant Biol. 9, 470–477. doi: 10.1016/j.pbi.2006.07.015 16890477

[B14] Dodd I. C. (2003). Hormonal interactions and stomatal responses. J. Plant Growth Regul. 22, 32–46. doi: 10.1007/s00344-003-0023-x

[B15] Farkas I. Dombradi V. Miskei M. Szabados L. Koncz C. (2007). *Arabidopsis* PPP family of serine/threonine phosphatases. Trend. Plant Sci. 12, 169–176. doi: 10.1016/j.tplants.2007.03.003 17368080

[B16] Fellner T. Lackner D. H. Hombauer H. Piribauer P. Mudrak I. Zaragoza K.. (2003). A novel and essential mechanism determining specificity and activity of protein phosphatase 2A (PP2A) in *vivo* . Genes Dev. 17, 2138–2150. doi: 10.1101/gad.259903 12952889 PMC196455

[B17] Hauser F. Li Z. Waadt R. Schroeder J. I. (2017). Snapshot: Abscisic acid signaling. Cell 171, 1708–1708.e1700. doi: 10.1016/j.cell.2017.11.045 29245015 PMC5895850

[B18] Horsch R. B. Fry J. E. Hoffmann N. L. Eichholtz D. Rogers S. G. Fraley R. T. (1985). A simple and general-method for transferring genes into plants. Science 227, 1229–1231. doi: 10.1126/science.227.4691.1229 17757866

[B19] Hu R. Zhu Y. Wei J. Chen J. Shi H. Shen G.. (2017). Overexpression of *PP2A-C5* that encodes the catalytic subunit 5 of protein phosphatase 2A in *Arabidopsis* confers better root and shoot development under salt conditions. Plant Cell Envir. 40, 150–164. doi: 10.1111/pce.12837 27676158

[B20] Huntingford C. Smith D. M. Davies W. J. Falk R. Sitchd S. Mercado L. M. (2015). Combining the [ABA] and net photosynthesis-based model equations of stomatal conductance. Ecol. Model. 300, 81–88. doi: 10.1016/j.ecolmodel.2015.01.005

[B21] Janssens V. Goris J. (2001). Protein phosphatase 2A: A highly regulated family of serine/threonine phosphatases implicated in cell growth and signalling. Biochem. J. 353, 417–439. doi: 10.1042/bj3530417 11171037 PMC1221586

[B22] Kosar F. Akram N. A. Ashraf M. (2015). Exogenously-applied 5-aminolevulinic acid modulates some key physiological characteristics and antioxidative defense system in spring wheat (*Triticum aestivum* L.) seedlings under water stress. South Afric. J. Bot. 96, 71–77. doi: 10.1016/j.sajb.2014.10.015

[B23] Kwak J. M. Moon J. H. Murata Y. Kuchitsu K. Leonhardt N. DeLong. A.. (2002). Disruption of a guard cell-expressed protein phosphatase 2A regulatory subunit, RCN1, confers abscisic acid insensitivity in *Arabidopsis* . Plant Cell 14, 2849–2861. doi: 10.1105/tpc.003335 12417706 PMC152732

[B24] Lefoulon C. Boeglin M. Moreau B. Very A. A. Szponarski W. Dauzat M.. (2016). The *Arabidopsis* AtPP2CA protein phosphatase inhibits the GORK K^+^ efflux channel and exerts a dominant suppressive effect on phosphomimetic-activating mutations. J. Biol. Chem. 291, 6521–6533. doi: 10.1074/jbc.M115.711309 26801610 PMC4813591

[B25] Li D. M. Zhang J. Sun W. J. Li Q. Dai A. H. Bai J. G. (2011). 5-Aminolevulinic acid pretreatment mitigates drought stress of cucumber leaves through altering antioxidant enzyme activity. Sci. Hortic. 130, 820–828. doi: 10.1016/j.scienta.2011.09.010 21353326

[B26] Li Y. Z. Zhao Z. Q. Song D. D. Yuan Y. X. Sun H. J. Zhao J. F.. (2021). SnRK2.6 interacts with phytochrome B and plays a negative role in red light-induced stomatal opening. Plant Signal. Behav. 3, 16(6):1913307. doi: 10.1080/15592324.2021.1913307 PMC8143258 33853508

[B27] Liu D. Wu L. Naeem M. S. Liu H. Deng X. Xu L.. (2013). 5-Aminolevulinic acid enhances photosynthetic gas exchange, chlorophyll fluorescence and antioxidant system in oilseed rape under drought stress. Acta Physiol. Plant 35, 2747–2759. doi: 10.1007/s11738-013-1307-9

[B28] Liu L. B. Xiong L. J. An Y. Y. Zheng J. Wang L. J. (2016). Flavonols induced by 5-aminolevulinic acid are involved in regulation of stomatal opening in apple leaves. Hortic. Plant J. 2, 323–330. doi: 10.1016/j.hpj.2017.02.002

[B29] Livak K. J. Schmittgen T. D. (2010). Analysis of relative gene expression data using real-time quantitative PCR and the 2^-ΔΔCT^ method. Methods 25, 402–408. doi: 10.1006/meth.2001.1262 11846609

[B30] Mathe C. Garda T. Freytag C. M-Hamvas M. (2019). The role of serine-threonine protein phosphatase PP2A in plant oxidative stress signaling-facts and hypotheses. Int. J. Mol. Sci. 20 (12), 3028. doi: 10.3390/ijms20123028 31234298 PMC6628354

[B31] Murata Y. Mori I. C. Munemasa S. (2015). Diverse stomatal signaling and the signal integration mechanism. Annu. Rev. Plant Biol. 66, 369–392. doi: 10.1146/annurev-arplant-043014-114707 25665132

[B32] Mustilli A. C. Merlot S. Vavasseur A. Fenzi F. Giraudat J. (2002). *Arabidopsis* OST1 protein kinase mediates the regulation of stomatal aperture by abscisic acid and acts upstream of reactive oxygen species production. Plant Cell 14, 3089–3099. doi: 10.1105/tpc.007906 12468729 PMC151204

[B33] Pan J. H. (2021). Lowering the carbon emissions peak and accelerating the transition towards net zero carbon. Chin. J. Urban Environ. Stud. 9, 215001. doi: 10.1142/S2345748121500135

[B34] Pernas M. Garcia-Casado G. Rojo E. Solano R. Sanchez-Serrano J. J. (2007). A protein phosphatase 2A catalytic subunit is a negative regulator of abscisic acid signalling. Plant J. 51, 763–778. doi: 10.1111/j.1365-313X.2007.03179.x 17617176

[B35] Saito N. Munemasa S. Nakamura Y. Shimoishi Y. Mori I. C. Murata Y. (2008). Roles of RCN1, regulatory A subunit of protein phosphatase 2A, in methyl jasmonate signaling and signal crosstalk between methyl jasmonate and abscisic acid. Plant Cell Physiol. 49, 1396–1401. doi: 10.1093/pcp/pcn106 18650210

[B36] Šantrůček J. Hronková M. Květoň J. Sage R. F. (2003). Photosynthesis inhibition during gas exchange oscillations in ABA-treated *Helianthus annuus*: relative role of stomatal patchiness and leaf carboxylation capacity. Photosynthetica 41, 241–252. doi: 10.1023/B:PHOT.0000011957.57326.5e

[B37] Wang Y. Guo C. H. Chen X. J. Jia L. Q. Guo X. N. Chen R. S.. (2021). Carbon peak and carbon neutrality in China: Goals, implementation path and prospects. China Geol. 4, 720–746. doi: 10.31035/cg2021083

[B38] Wang L. J. Jiang W. B. Huang B. J. (2004). Promotion of 5-aminolevulinic acid on photosynthesis of melon (*Cucumis melo*) seedlings under low light and chilling stress conditions. Physiol. Plant 121, 258–264. doi: 10.1111/j.0031-9317.2004.00319.x 15153193

[B39] Wang L. J. Jiang W. B. Zhang Z. Yao Q. H. Matsui H. Ohara H. (2003). Biosynthesis and physiological activities of 5-aminolevulinic acid (ALA)and its potential application in agriculture. Plant Physiol. Commun. 39, 185–192. doi: 10.13592/j.cnki.ppj.2003.03.001

[B40] Watkins J. M. Hechler P. J. Muday G. K. (2014). Ethylene-induced flavonol accumulation in guard cells suppresses reactive oxygen species and moderates stomatal aperture. Plant Physiol. 164, 1707–1717. doi: 10.1104/pp.113.233528 24596331 PMC3982735

[B41] Wu Y. Liao W. B. Dawuda M. M. Hu L. L. Yu J. H. (2019). 5-Aminolevulinic acid (ALA) biosynthetic and metabolic pathways and its role in higher plants: a review. Plant Growth Regul. 87, 357–374. doi: 10.1007/s10725-018-0463-8

[B42] Xiong L. J. An Y. Y. Wang L. J. (2018). The Role of microtubule skeleton and PP1/PP2A protein phosphatase in ALA-ABA regulating stomatal movement in apple leaves. Acta Hortic. Sin. 45, 2073–2088. doi: 10.16420/j.issn.0513-353x.2018-0134

[B43] Xu M. Li H. Liu Z. N. Wang X. H. Xu P. Dai S. J.. (2021). The soybean CBL-interacting protein kinase, GmCIPK2, positively regulates drought tolerance and ABA signaling. Plant Physiol. Bioch. 167, 980–989. doi: 10.1016/j.plaphy.2021.09.026 34583133

[B44] Yao J. N. (2021). China’s biomass energy utilization potential is about 460 million tons of standard coal. China Energy News 019. doi: 10.28693/n.cnki.nshca.2021.002202

[B45] Yoshida R. Hobo T. Ichimura K. Mizoguchi T. Takahashi F. Aronso J.. (2002). ABA-activated SnRK2 protein kinase is required for dehydration stress signaling in *Arabidopsis* . Plant Cell Physiol. 43, 1473–1483. doi: 10.1093/pcp/pcf188 12514244

[B46] Yu F. Wu Y. Xie Q. (2015). Precise protein post-translational modifications modulate ABI5 activity. Trends Plant Sci. 20, 569–575. doi: 10.1016/j.tplants.2015.05.004 26044742

[B47] Zhang J. H. Jia W. S. Yang J. C. Ismail A. M. (2006). Role of ABA in integrating plant responses to drought and salt stresses. Field Crop Res. 97, 111–119. doi: 10.1016/j.fcr.2005.08.018

[B48] Zhang Z. P. Yao Q. H. Wang L. J. (2011). Expression of yeast *Hem1* controlled by *Arabidopsis HemA1* promoter enhances leaf photosynthesis in transgenic tobacco. Mol. Biol. Rep. 38, 4369–4379. doi: 10.1007/s11033-010-0564-6 21110104

