# Supplementary material for: ALA induces stomatal opening through regulation among PTPA, PP2AC, and SnRK2.6
Source: Front Plant Sci. 2023 Aug 30;14:1206728. doi: 10.3389/fpls.2023.1206728 (PMC10499497; doi:10.3389/fpls.2023.1206728)
Supplement: Supplementary file 1 [file DataSheet_1.zip › All supplementary materials/Appendix 3 The map of the pBridge vector.pdf]

pBridge™ Vector Information PT3212-5

GenBank Accession No.: Submission in progress. Cat. No. 630404

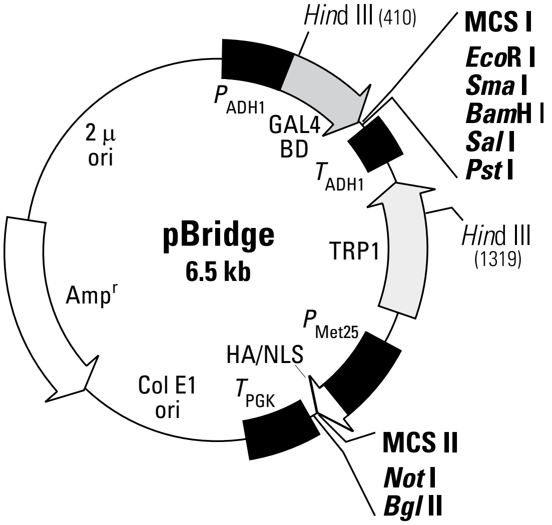

MCS I:

MATCHMAKER 5' DNA-BD Insert Screening Amplimer & GAL4 DNA-BD Sequencing Primer →

GAL4 DNA-BD →

827
TCA TCG GAA GAG AGT AGT AAC AAA GGT CAA AGA CAG TTG ACT GTA TCG CCG

878
GAA TTC CCG GGG ATC CGT CGA CCT GCA GCC AAG CTA ATT CCG GGC GAA TTT

*Eco*R I (GAA TTC), *Sma* I (CCC GGG), *Bam*H I (GGA TCC), *Sal* I (GTC GAC), *Pst* I (CTG CAG), STOP (ORF 2) (TAA)

929
CTT ATG ATT TAT GAT TTT TAT TAT TAA ATA AGT TAT AAA AAA AAT AAG TGT ATA

STOP (ORF 3) (TGA), STOP (ORF 1) (TAA)

983
CAA ATT TTA AAG TGA CTC TTA GGT TTT AAA ACG

← MATCHMAKER 3' DNA-BD Insert Screening Amplimer

MCS II:

2682
AAG AAG AGA AAG GTG GCG GCC GCA TTA GCC CGA AGA TCT TCG GGC TGA

Not I (GCG GCC GC), *Bgl* II (AGA TCT), STOP (TGA)

Restriction map and multiple cloning sites of pBridge Vector. Unique restriction sites are in bold.

Description:

pBridge™ expresses two proteins: a DNA-binding domain fusion, and an additional protein (1–3). pBridge thus allows establishment of three-hybrid systems when used in combination with an activation domain fusion vector and yeast strains from any of Clontech's GAL4-based two-hybrid systems, including Matchmaker™ Gold. This vector generates a hybrid protein that contains the sequences for the GAL4 DNA-binding domain (DNA-BD; a.a. 1–147) and the sequence cloned into MCS I. The fusion protein is expressed in yeast host cells from the constitutive *ADH1* promoter; transcription is terminated at the *ADH1* transcription termination signal. The hybrid protein is targeted to the yeast nucleus by nuclear localization sequences (NLS) that are an intrinsic part of the GAL4 DNA-BD (3). An additional gene of interest can be cloned into MCS II which is located downstream of an HA epitope and a second NLS. The resulting fusion protein is conditionally expressed from the *MET25* promoter in response to methionine levels in the medium; i.e., it is repressed in the presence of 1 mM methionine and expressed in the absence of methionine (1).

(PR0Z3760)

Clontech

United States/Canada
800.662.2566
Asia Pacific
+1.650.919.7300
Europe
+33.(0)1.3904.6880
Japan
+81.(0)77.543.6116

Clontech Laboratories, Inc.
A Takara Bio Company
1290 Terra Bella Ave.
Mountain View, CA 94043
Technical Support (US)
E-mail: tech@clontech.com
www.clontech.com

pBridge is a shuttle vector that replicates autonomously in both *E. coli* and *S. cerevisiae*. It carries the *bla* gene (for ampicillin resistance in *E. coli*) and the *TRP1* nutritional marker that allow yeast auxotrophs carrying pBridge to grow on limiting synthetic medium lacking tryptophan. **Note:** Yeast strain Y187 is a methionine auxotroph; therefore, haploid Y187 harboring pBridge cannot be grown on medium lacking methionine.

Use:

The pBridge Vector now makes it possible to investigate ternary protein complex formation (1, 2). pBridge contains two distinct multiple cloning sites to allow expression of the DNA-BD fusion as well as a third protein. When pBridge is used in conjunction with the AD fusion vector and yeast strains from any one of Clontech's GAL4-based two-hybrid systems, a 'three-hybrid' system can be established hat is dependent on the expression of a third protein.

The yeast two-hybrid system has proven to be a powerful molecular approach for detecting protein-protein (binary) interactions and has contributed significantly to the dissection of many molecular pathways. However, the *two*-hybrid system is designed to detect the interaction between just two proteins, which are expressed as the AD and BD fusions.

The figure below demonstrates the more complex protein interactions that can be investigated with the three-hybrid system. The third protein in this system can participate in the interaction in several ways: as a "bridge", interacting with two proteins that do not directly interact with each other; to stabilize a weak interaction between two proteins; or as an inhibitor or modifier (e.g., kinase; 3) of one or both of the proteins. Alternatively, a competitor of a two-hybrid interaction can be expressed from this promoter to confirm the specificity of the two-hybrid interaction (1). The conditional expression of the third protein allows investigation of its role in the interaction between the AD and BD fusion proteins. Like the two-hybrid system, the three-hybrid system can be used to screen libraries to clone new interacting partners, either for the third protein or the binding domain fusion.

Expression of the third protein is controlled by a conditional methionine promoter (P_{MET25}) such that it is expressed in the absence of methionine. This allows expression to be switched on or off by a simple replica plating step. The effect of the third protein is indicated by expression of a reporter or nutritional marker.

To facilitate experiments with pBridge, we offer three drop-out media supplements that lack methionine: –His/–Leu/–Met/–Trp (Cat No. 630429); –Leu–Met/–Trp (Cat. No. 630430); and –Met/–Trp (Cat. No. 630431).

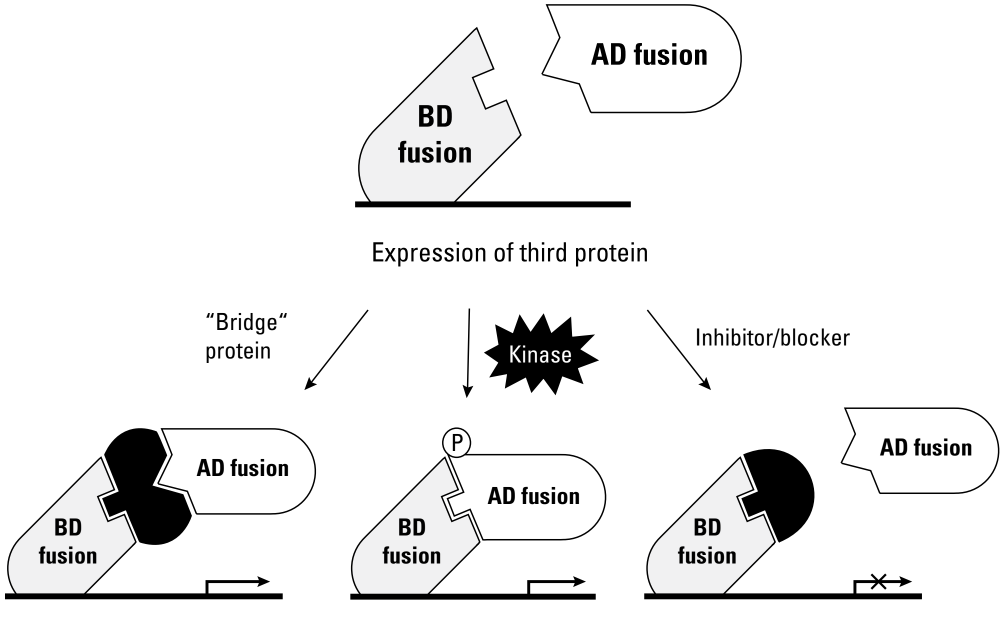

The three-hybrid system. pBridge expresses both the DNA-BD fusion and the third protein. The activation domain fusion is expressed from a separate two-hybrid system vector. The conditionally expressed third protein can play a structural (left), modifying (center), or inhibitory (right) role in the interaction that restores reporter gene expression.

Location of features:
- Promoter
  Fragment containing the *S. cerevisiae ADH1* promoter: 10–406
- GAL4 DNA-binding domain polypeptide
  Start codon: 434–436; stop codon: 953–955
  GAL4 codons 1–147: 434–874
- MCS I: 878–905
- Transcription termination signal
  Fragment carrying the *S. cerevisiae ADH1* terminator: 921–1112
- *TRP1* coding sequence
  Start codon: 1835–1833; stop codon: 1163–1161
- *S. cerevisiae MET25* promoter: 2117–2609
- HA epitope and nuclear localization sequence: 2610–2699
- MCS II: 2700–2723
- *S. cerevisiae PGK* terminator: 2733–3120
- Col E1 origin of replication: 3324–3967
- Ampicillin resistance gene (β-lactamase): 5045–3968
  Promoter: 5045–5017
  Coding sequence: 4975–4115
- Fragment containing the 2 μ origin of replication: 5362–6526

Primer locations:
- Matchmaker DNA-BD 5' Insert Screening Amplimer (Cat. No. 5417-1) or GAL4 BD Sequencing Primer (Cat. No. 6474-1): 827—843
- Matchmaker DNA-BD 3' Insert Screening Amplimer (Cat. No. 5417-1): 1015–994

Propagation in *E. coli:*
- Suitable host strains: DH5α and other general purpose strains.
- Selectable marker: plasmid confers resistance to ampicillin (50 μg/ml) to *E. coli* hosts.
- *E. coli* replication origin: Col E1
- Copy number: 15–20

Propagation in *S. cerevisiae:*
- Suitable host strains: Y187(α)*, Y190(a), HF7c(a), CG1945(a), PJ69-2A(a), PJ69-4A(a)*, SFY526(a), AH109(a) and Y2H Gold.

  Note: When using pBridge, yeast strains need to be streaked 3 times to single colony on agar plates lacking Methionine (SD/-Met) since they tend to lose tolerance to this medium if not maintained on it routinely.
- Selectable marker: *TRP1*
- *S. cerevisiae* replication origin: 2 μ
- Copy number: multiple copy

References:

1. Tirode, F., *et al.* (1997) *J. Biol. Chem.* **272**:22995–22999.
2. Brachmann, R. & Boeke, J. (1997) *Curr. Opin. Biotechnol.* **8**:561–568.
3. Osborne, M., *et al.* (1995) *Biotechnology* **13**:1474–1478.

*These strains are methionine auxotrophs; protein cannot be expressed from MCS II of pBridge in these strains. However, these strains may be used when mating to a methionine autotroph.

Note:The attached sequence file has been compiled from information in the sequence databases, published literature, and other sources, together with partial sequences obtained by Clontech. This vector has not been completely sequenced.

Notice to Purchaser

Clontech products are to be used for research purposes only. They may not be used for any other purpose, including, but not limited to, use in drugs, *in vitro* diagnostic purposes, therapeutics, or in humans. Clontech products may not be transferred to third parties, resold, modified for resale, or used to manufacture commercial products or to provide a service to third parties without written approval of Clontech Laboratories, Inc.

Clontech, the Clontech logo and all other trademarks are the property of Clontech Laboratories, Inc., unless noted otherwise. Clontech is a Takara Bio Company. ©2010 Clontech Laboratories, Inc.
