# Supplementary material for: ALA induces stomatal opening through regulation among PTPA, PP2AC, and SnRK2.6
Source: Front Plant Sci. 2023 Aug 30;14:1206728. doi: 10.3389/fpls.2023.1206728 (PMC10499497; doi:10.3389/fpls.2023.1206728)
Supplement: Supplementary file 1 [file DataSheet_1.zip › All supplementary materials/Supplementary figures.docx]

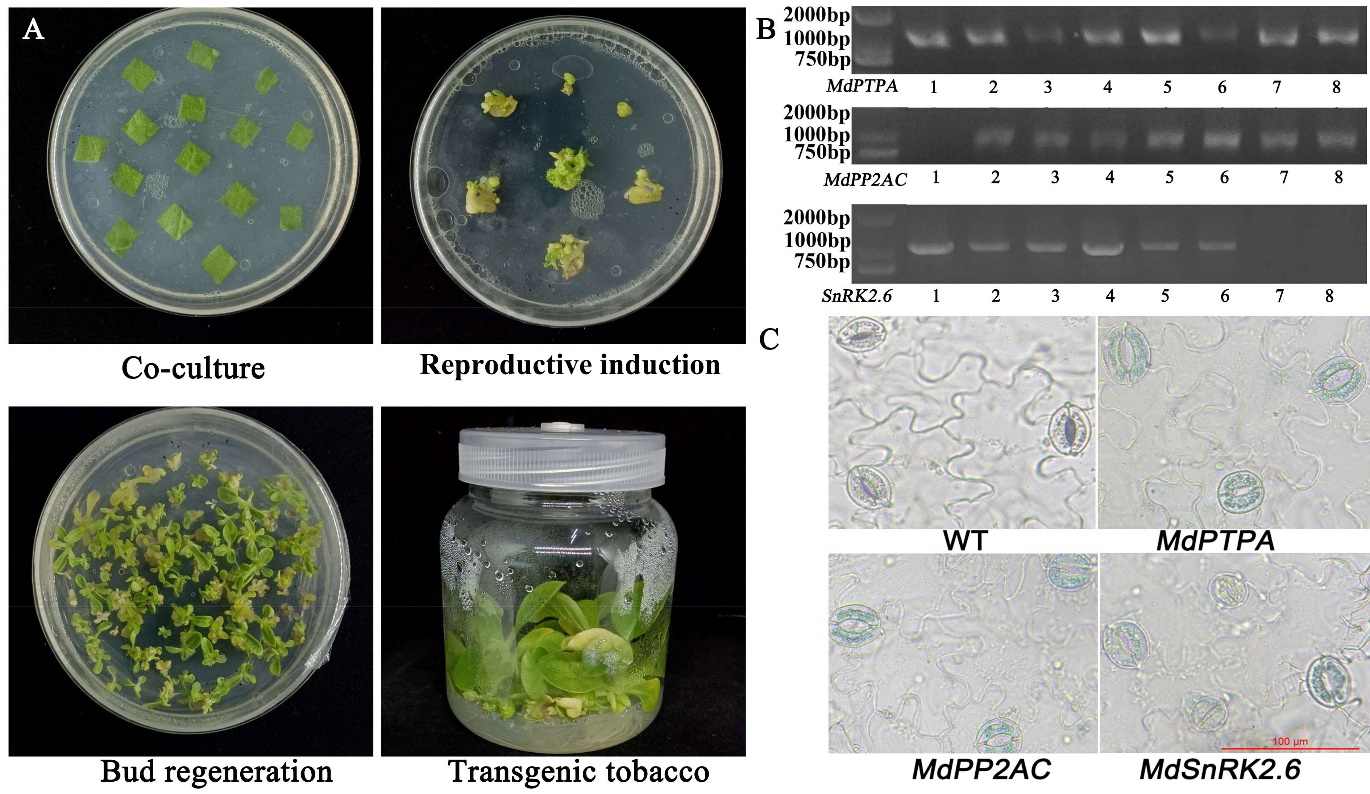


Fig. S1 Identification of the transgenic plants which overexpressed *MdPTPA*, *MdPP2AC* and *MdSnRK2.6*, respectively, in tobacco (*Nicotiana benthamiana* Domin). A: Genetic transformation of *MdPTPA*, *MdPP2AC* and *MdSnRK2.6* into tobacco by the leaf disc method with *Agrobacterium tumefaciens* GV3101 as a vector to obtain stable transgenic plants. B: PCR detection of the transgenic tobacco expressing *MdPTPA*, *MdPP2AC* and *MdSnRK2.6*, respectively, where the number above the gel bands are different plantlet lines. (C) GUS detection of *MdPTPA*, *MdPP2AC* and *MdSnRK2.6* expressions in the transgenic tobaccos. The green dying in the guard cell shows *MdPTPA*, *MdPP2AC* and *MdSnRK2.6*: GUS expressions in the transgenic plants. Scale bar = 100 µm.


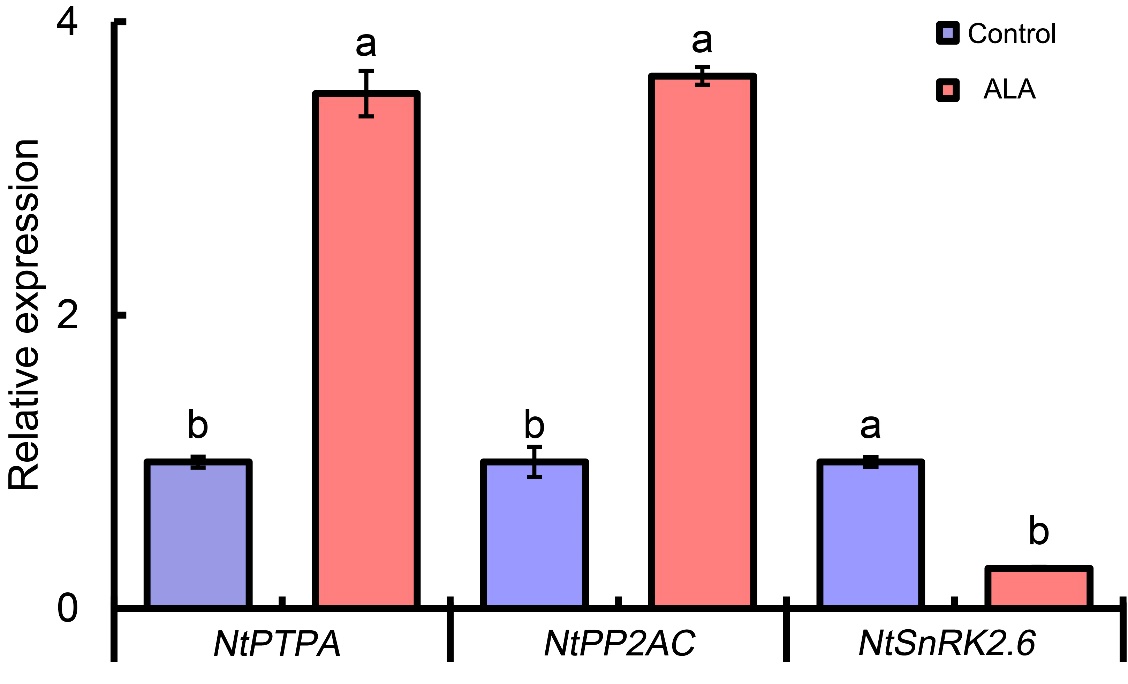


Fig. S2 The qRT-PCR detection of *NtPTPA*, *NtPP2AC* and *NtSnRK2.6* expressions in the tobacco plants, respectively, treated with ALA or not. During the experiments, the isolated epidermal strips of tobacco were incubated at 25℃ in MES-KCl buffer without plant hormones (Control). After 2 h illumination pretreatment (240 µmol m^−2^ s^−1^), the strips were transferred into the same buffer but containing 0.5 mg L^−1^ ALA and illuminated one hour more, then the RNA was extracted for qRT-PCR. The values are the means ± SE of three biological replicates and the different letters represent significant difference at *P* = 0.05.


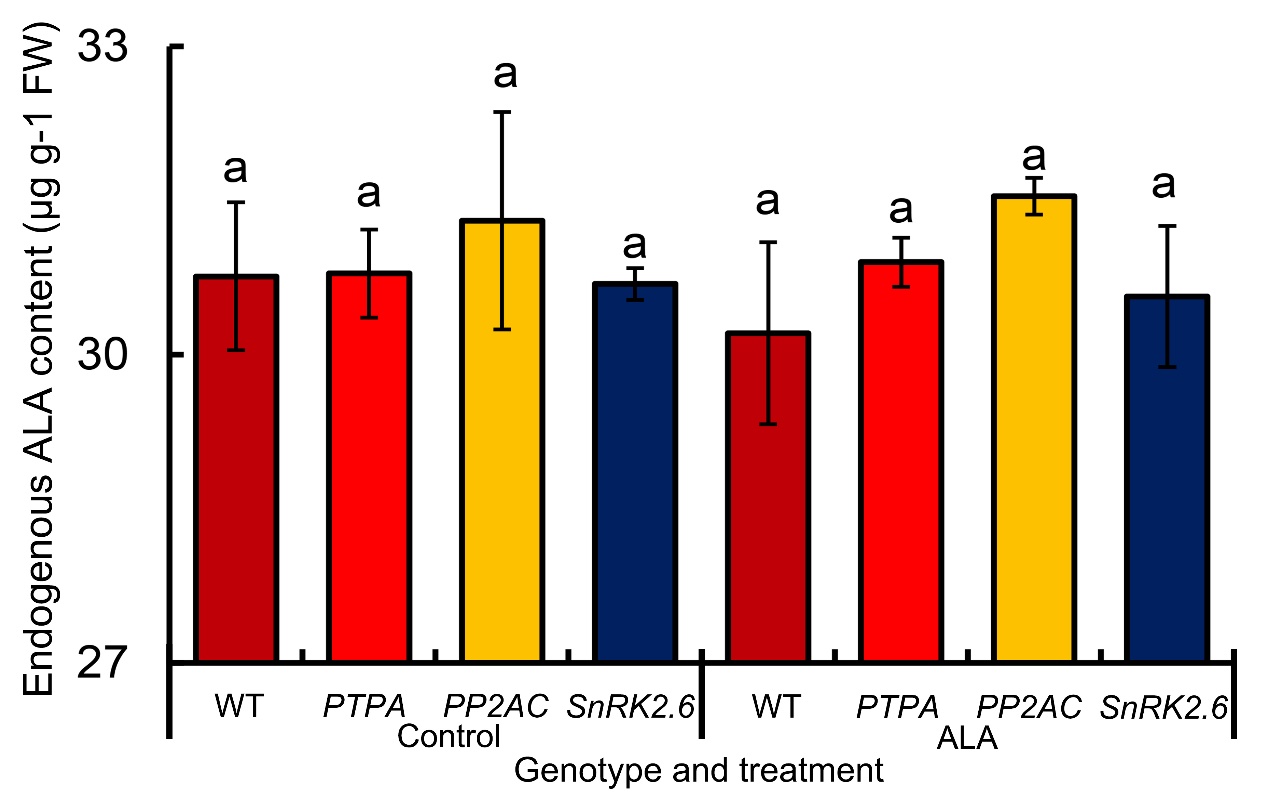


Fig. S3 Endogenous ALA content of different transgenic tobaccos. The data present the means of three independent replicates.


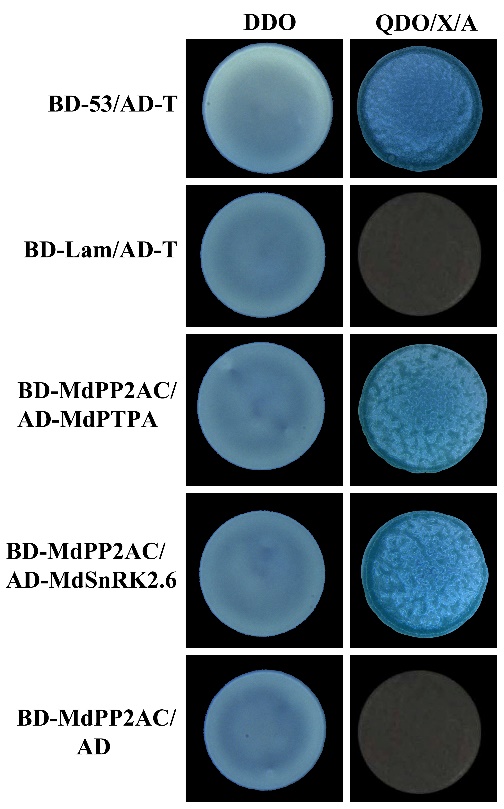


Fig. S4 Interactions of MdPP2AC with MdPTPA and MdSnRK2.6. Yeast two-hybrid (Y2H) assay showed that MdPP2AC interacted with MdPTPA and MdSnRK2.6. The combination of BD-53 plus AD-T was used as a positive control, and BD-Lam plus AD-T was used as a negative control, where BD was pGBKT7 vector, AD was pGADT7 vector, DDO was double dropout SD medium (lacking leucine and tryptophan) and QDO/X/A was quadruple dropout medium, SD/–Ade/–His/–Leu/–Trp supplemented with X-a-Gal and AbA.


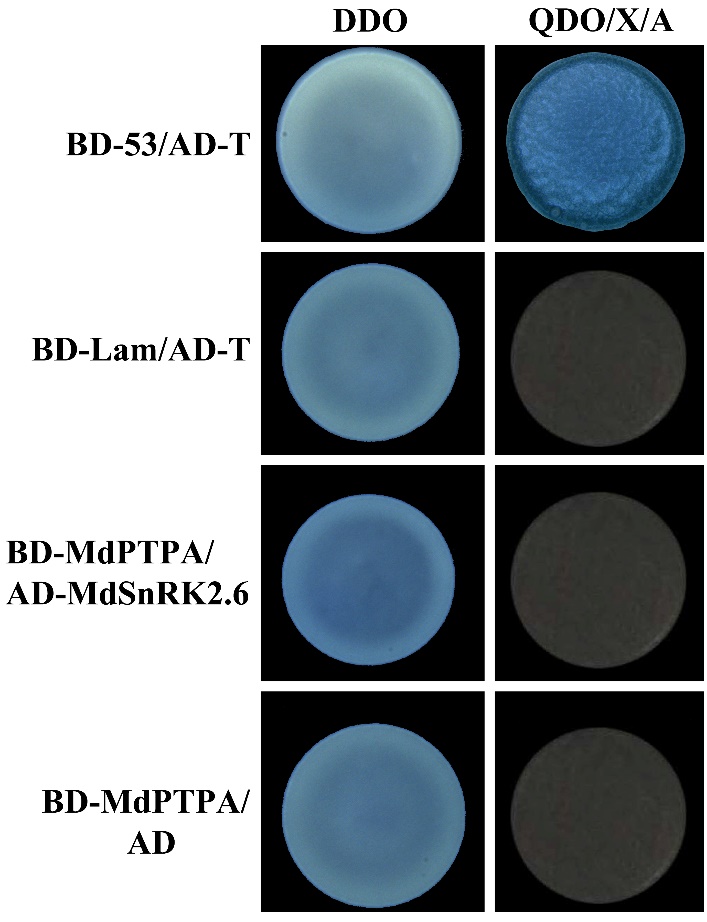


Fig. S5 Interactions of MdPTPA with MdSnRK2.6. Yeast two-hybrid (Y2H) assay showed that MdPTPA did not interacted with MdSnRK2.6. The combination of BD-53 plus AD-T was used as a positive control, and BD-Lam plus AD-T, BD-MdPTPA plus AD were used as a negative control, where BD was pGBKT7 vector, AD was pGADT7 vector, DDO was double dropout SD medium (lacking leucine and tryptophan) and QDO/X/A was quadruple dropout medium, SD/–Ade/–His/–Leu/–Trp supplemented with X-a-Gal and AbA.
